# PUFA-Derived *N*-Acylethanolamide Probes Identify Peroxiredoxins and Small GTPases as Molecular Targets in LPS-Stimulated RAW264.7 Macrophages

**DOI:** 10.1021/acschembio.1c00355

**Published:** 2022-07-22

**Authors:** Ian-Arris de Bus, Antoine H. P. America, Norbert C. A. de Ruijter, Milena Lam, Jasper W. van de Sande, Mieke Poland, Renger F. Witkamp, Han Zuilhof, Michiel G. J. Balvers, Bauke Albada

**Affiliations:** † Division of Human Nutrition and Health, Wageningen University & Research, Stippeneng 4, 6708 WE Wageningen, The Netherlands; ‡ Laboratory of Organic Chemistry, Wageningen University & Research, Stippeneng 4, 6708 WE Wageningen, The Netherlands; § Wageningen Plant Research, Business Unit Bioscience, Wageningen University & Research, Droevendaalsesteeg 1, 6708 PB Wageningen, The Netherlands; ∥ Laboratory of Cell Biology, Wageningen Light Microscopy Centre, Wageningen University & Research, Droevendaalsesteeg 1, 6708 PB Wageningen, The Netherlands; ⊥ School of Pharmaceutical Sciences and Technology, Tianjin University, 92 Weijin Road, 300072 Tianjin, People’s Republic of China; # Department of Chemical and Materials Engineering, Faculty of Engineering, King Abdulaziz University, 21589 Jeddah, Saudi Arabia

## Abstract

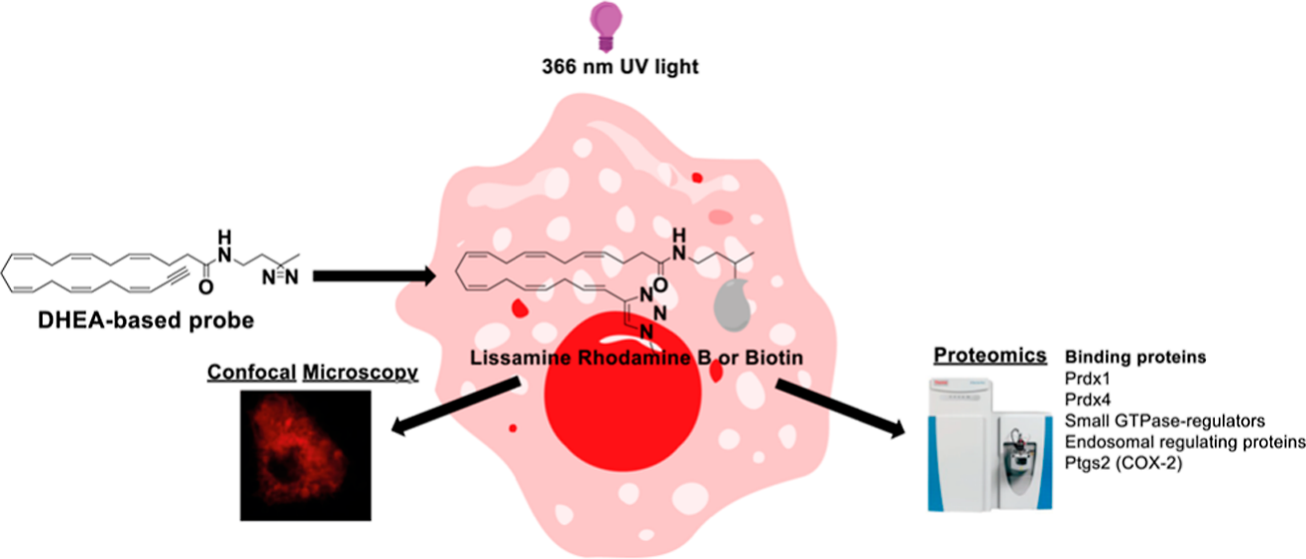

We studied the mechanistic and biological origins of anti-inflammatory poly-unsaturated fatty acid-derived *N*-acylethanolamines using synthetic bifunctional chemical probes of docosahexaenoyl ethanolamide (DHEA) and arachidonoyl ethanolamide (AEA) in RAW264.7 macrophages stimulated with 1.0 μg mL^–1^ lipopolysaccharide. Using a photoreactive diazirine, probes were covalently attached to their target proteins, which were further studied by introducing a fluorescent probe or biotin-based affinity purification. Fluorescence confocal microscopy showed DHEA and AEA probes localized in cytosol, specifically in structures that point toward the endoplasmic reticulum and in membrane vesicles. Affinity purification followed by proteomic analysis revealed peroxiredoxin-1 (Prdx1) as the most significant binding interactor of both DHEA and AEA probes. In addition, Prdx4, endosomal related proteins, small GTPase signaling proteins, and prostaglandin synthase 2 (Ptgs2, also known as cyclooxygenase 2 or COX-2) were identified. Lastly, confocal fluorescence microscopy revealed the colocalization of Ptgs2 and Rac1 with DHEA and AEA probes. These data identified new molecular targets suggesting that DHEA and AEA may be involved in reactive oxidation species regulation, cell migration, cytoskeletal remodeling, and endosomal trafficking and support endocytosis as an uptake mechanism.

## Introduction

Poly-unsaturated fatty acids (PUFAs) are essential lipids for human development and functioning, and they support important roles such as immune regulation.^1^ Besides PUFAs, their corresponding amides, esters, and ethers also possess immunoregulatory activities.^1−3^ Currently, the prototypical endocannabinoid arachidonoyl ethanolamide (AEA, a.k.a. anandamide), the ethanolamine conjugate of arachidonic acid (AA) (20:4*n*-6), has well-described interactions with cannabinoid (CB) and other receptors^1,4−7^ and is known to be converted to inflammatory-regulating prostamides.^1,5^ Nevertheless, the full spectrum of its uptake and biological mechanisms underlying its effects are not yet fully understood.^1,8^ An important structural analogue of AEA is the *n*-3 PUFA amide docosahexaenoyl ethanolamide (DHEA), the ethanolamine conjugate of docosahexaenoic acid (DHA) (22:6*n*-3). Studies on lipopolysaccharide (LPS)-stimulated mouse-derived RAW264.7 macrophages and microglia cell lines showed that DHEA reduced the formation of nitric oxide (NO), COX-2-derived prostaglandins, and thromboxanes and also lowered expression and production of various inflammatory-regulating cytokines such as monocyte chemoattractant protein-1 (MCP-1), interleukin-6 (IL-6), tumor necrosis factor alpha (TNFα), and IL-1β.^9,10^ Apart from cytokine regulation in macrophages, DHEA exerted synaptogenic and neuroprotective effects in neural cells,^10,11^ stimulated reactive oxidation species (ROS) production in head and neck squamous cell carcinoma (HNSCC) cells,^12^ and reduced inflammatory and nociceptive pain-related behavior in mice.^13^ Additionally, DHEA and AEA are metabolized by COX-2, 15-LOX, and CYP450 to yield compounds with distinct anti-inflammatory and antitumorigenic properties.^1,2,14−18^ Other studies reported interactions between DHEA and the cannabinoid receptors CB_1_/CB_2_, transient receptor potential V1 (TRPV-1), and peroxisome proliferator-activated receptors (PPARs), although the obtained agonistic effects seem to depend on the model that was used.^1,9,14,16,19−21^ Our previous studies on LPS-stimulated RAW264.7 macrophages did not show DHEA agonism with CB_1_/CB_2_ or PPARs but rather indicated an important role in reducing COX-2-derived prostaglandins.^9,17^ Clearly, many open questions persist about DHEA signaling in LPS-stimulated macrophages.

**Figure 1:**
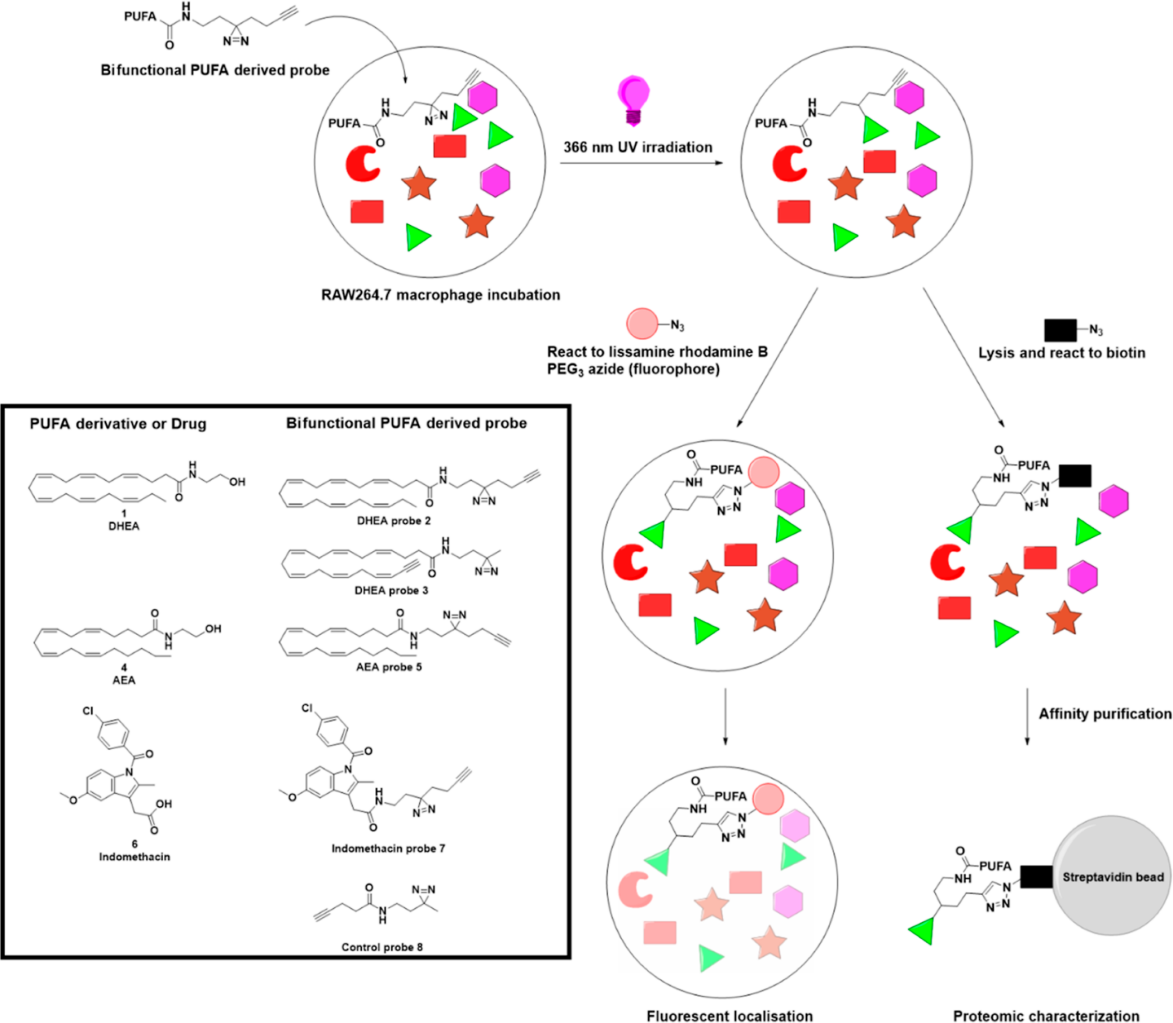
Schematic application of bifunctional PUFA-derived probes for cellular visualization and proteomic characterization. First, the diazirine group is covalently linked to the molecular (protein) interaction partner(s) when exposed to 366 nm UV irradiation. Then, the PUFA-derived probes are either (i) visualized using CuAAC coupling to a fluorophore or (ii) purified by affinity purification with streptavidin beads and biotinylated probes. Affinity-based purification is followed by tryptic digestion and MS/MS-based characterization. Chemical structures of the natural PUFA derivatives or drugs, and their synthetic derived bifunctional probes, are shown in the figure.

In the current study, we aim to elucidate underlying mechanisms of the anti-inflammatory effects of AEA and DHEA in 1.0 μg mL^–1^ LPS-stimulated murine RAW264.7 macrophages by applying novel bifunctional PUFA-derived probes (Figure 1). These probes contain a 366 nm UV-active diazirine moiety to covalently attach to their interaction targets and contain a terminal alkyne to selectively purify or visualize the chemical probes using a copper-mediated alkyne–azide click reaction (CuAAC) with biotin or a fluorescent probe, respectively (Figure 1).^1,22,23^ Previously, similar methodological setups identified protein interactions with arachidonoyl, oleoyl, palmitoyl, and stearoyl probes in human HEK293T and mouse Neuro2a cells, which led to new druggable sites in the endocannabinoid system.^6^ Similarly, nonsteroidal anti-inflammatory drug (NSAID) probes^24^ were used to unravel the interaction of celecoxib with prostaglandin E synthase.^25^

To characterize new protein interaction partners of DHEA and AEA in LPS-stimulated RAW264.7 macrophages, we first assessed whether our probes displayed comparable biological effects as their parent compounds. Subsequently, we studied their localization in the macrophages by confocal fluorescence microscopy. Affinity purification followed by proteomic analysis enabled analysis of the protein interactome and provided insights into the molecular interaction partners and underlying pathways. Our data confirm previously proposed roles of both AEA and DHEA in the regulation of ROS production, cytoskeletal remodeling, and migration, which we attribute to newly uncovered interactions with peroxiredoxins (Prdxs) and small GTPase signaling proteins.

## Methods

### Cell Culture

All cell experiments were performed in RAW264.7 macrophages (American Type Culture Collection) cultured in Dulbecco’s modified Eagle’s medium (DMEM) containing 10% fetal calf serum (FCS) and 1% penicillin and streptomycin (P/S). Cells were incubated at 37 °C and 5% CO_2_ in a humidified incubator.

### Cytotoxicity and Anti-inflammatory Effects

Macrophages were seeded at 2.5 × 10^5^ cells mL^–1^ and incubated overnight in 24-well plates (Corning Life Sciences) containing 0.5 mL of the medium per well. The medium of adherent cells was discarded and replaced with a fresh medium containing 5 or 10 μM compounds (in 0.1% v/v EtOH for PUFA conjugates or in 0.1% v/v DMSO for indomethacin) or a vehicle (0.1% v/v EtOH or 0.1% v/v DMSO) control. Cells were preincubated with the compounds for 30 min before stimulation with 1.0 μg mL^–1^ LPS in 0.1% phosphate-buffered saline (PBS) or 0.1% PBS control. After LPS addition, cells were incubated for 24 h in the dark (covered with an aluminum foil) to protect the probe from incidental UV exposure. Finally, the medium was collected, and IL-6, PGE_2_, and LDH concentrations were quantified as described in the Supporting Information.

### Proteomic Experiment

In 100 mm culture dishes, RAW264.7 macrophages were seeded at a density of (0.5–1.0) × 10^6^ cells mL^–1^ in 15 mL of the medium. After overnight culture, cells were stimulated with 5 mL of the fresh medium containing 1.0 μg mL^–1^ LPS in 0.1% PBS. After 4 h of the LPS stimulation, cells were incubated with 5 mL of the fresh medium containing 1.0 μg mL^–1^ LPS in 0.1% PBS and 10 μM synthetic probes in 0.1% EtOH or DMSO. Probe-treated macrophages were incubated for 4 h in the dark (covered with an aluminum foil). Following incubation, the medium and nonadherent cells were removed, after which the samples were placed on ice. Illumination with UV light was performed for 10 min at 366 nm and 1 mJ cm^–2^ with an UVP-C1000 crosslinker equipped with five 8 W light bulbs (Supporting Information, Figure 1) or under normal lamp light as control. After light treatment, cells were collected by scraping in 5 mL of the ice cold 1× PBS and used in the proteomic workup (detailed protocol is provided in the Supporting Information).

### Fluorescence and Immunostaining

Immunostaining and additional fluorescence click labeling were based on an existing protocol from the study by Gaebler et al.^26^ RAW264.7 macrophages were seeded in Ibidi μ-Slide 8-well ibiTreat polymer coverslips (Ibidi GmbH) with a density of 2.5 × 10^5^ cells mL^–1^, containing 300 μL of the cell suspension per well. Cells were allowed to grow overnight and then prestimulated for 4 h with 1.0 μg mL^–1^ LPS prior to a 4 h incubation with a fresh medium containing 10 μM probe or 0.1% EtOH (vehicle) and 1.0 μg mL^–1^ LPS (LPS prestimulation). Alternatively, cells were directly incubated with 10 μM probes or vehicle together with 1.0 μg mL^–1^ LPS for 4 h (no LPS prestimulation). After incubation, the medium of adherent cells was discarded, and cells were irradiated at 366 nm for 5 min on ice (lamp conditions in probe incubation) or placed on ice under “control” (normal lamp light) conditions. Immunostaining and fluorescence click labeling were performed as described in the Supporting Information.

### Statistical Analysis

LDH cytotoxicity, PGE_2_ ELISA, and IL-6 ELISA samples were measured in three separate experiments containing two technical replicates. Cytotoxicity values were presented as percentages and normalized to 1% Triton X-100 (100% toxicity) and vehicle (0.1% EtOH or DMSO) control (0% toxicity). Cytotoxicity values, PGE_2_ concentrations, and IL-6 concentrations are presented as mean with the standard deviation (SD). Graphical presentation and statistical analysis using one-way analysis of variance (ANOVA) with Dunnett’s multiple comparison post-hoc or Tukey’s multiple comparison post-hoc were performed in GraphPad Prism v 5.0.

## Results and Discussion

The bifunctional PUFA amide-derived probes (Figure 1) contain a photo-activatable diazirine for covalent attachment after 366 nm UV light treatment and a terminal alkyne for CuAAC-based affinity purification or labeling with a fluorescent group. Diazirine was introduced at the N-acyl end of PUFA where it likely did not interfere with the biological activity of PUFA-derived amides.^6^ Probes **2** and **3** are synthetic derivatives of DHEA **1**, in which probe **2** has alkyne at the *N*-acyl end and probe **3** has alkyne at the PUFA tail. Probe **5** is a synthetic mimic of the *n*-6 PUFA derivative AEA **4**, having alkyne and diazirine at the *N*-acyl end of the molecule; probe **7** is a previously reported NSAID probe of indomethacin **6**(24) and was used as a positive control,^24^ and probe **8** is a short pentynoyl-derived negative control probe lacking immunological effects. Identified interactions with control probe **8** are used to filter out nonspecific PUFA-derived probe interactions in the data analysis of the proteomics. Following earlier in vitro studies investigating the anti-inflammatory effect of DHEA and AEA in this and similar models,^9,12,14,15,17,18,21,27^ probe concentrations of 10 μM were used, which are of the same order of magnitude or even below those used in comparable studies with similar lipid probes.^6,28,29^ Lastly, applying 10 μM probe counters the attrition that is associated with different steps in the methodology (i.e., uptake of probes by macrophages, nonquantitative yield of photolabeling,^22^ and loss during enrichment) to obtain sufficient levels of labeled protein above the detection limit of the liquid chromatography tandem mass spectrometry (LC–MS/MS).

### Probes Reduce PGE_2_ and IL-6 Concentrations

We verified that our probes have similar biological effects to their parent compounds by measuring the production of PGE_2_ and IL-6 as well as the cytotoxicity of the chemical probes in 1.0 μg mL^–1^ LPS-stimulated RAW264.7. No significant cytotoxicity was observed for the PUFA-derived probes, and only for indomethacin probe **7** limited cytotoxicity was observed when compared to the vehicle control (Supporting Information, Figure 2 andTable 2).

**Figure 2:**
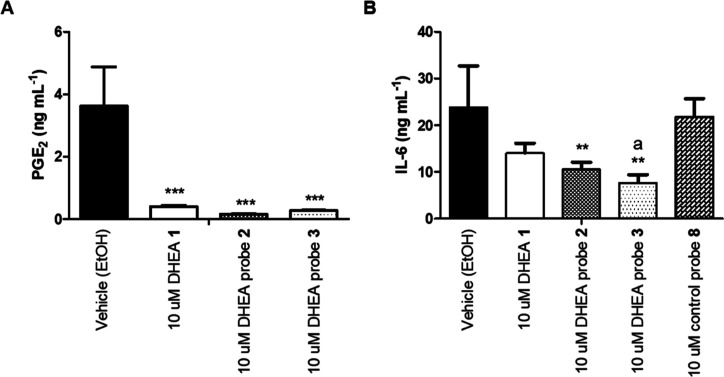
Medium concentration of inflammatory regulators released from 1.0 μg mL^–1^ LPS-stimulated RAW264.7 macrophages, incubated with 10 μM DHEA **1**, 10 μM DHEA-derived synthetic probe **2** or **3**, or 10 μM negative control probe **8**. (A) Medium concentrations of PGE_2_. (B) Medium concentrations of IL-6. Bars represent mean with SD (*N* = 3, technical duplicates). * indicates significant differences from the vehicle (EtOH) control (one-way ANOVA, Dunnett’s multiple comparison post-hoc; *P < 0.05, **P < 0.01, ***P < 0.001). “a” indicates significance between 10 μM DHEA **1** and 10 μM DHEA probe **3** incubation (one-way ANOVA, Tukey’s multiple comparison post-hoc; *P* < 0.05).

Incubation with 10 μM DHEA reduced PGE_2_ levels in the medium to 89% compared to those in the vehicle control [from 3.62 ± 1.26 ng mL^–1^ to 0.40 ± 0.10 ng mL^–1^ (*P* < 0.001)], and incubation with 10 μM synthetic DHEA probe **2** or **3** reduced PGE_2_ levels to 96% [0.16 ± 0.03 ng mL^–1^ (*P* < 0.001)] or to 93% [0.27 ± 0.05 ng mL^–1^ (*P* < 0.001)], respectively (Figure 2a and Supporting Information, Table 2). Similarly, incubation with 10 μM indomethacin or indomethacin probe **7** reduced PGE_2_ concentrations in the medium to levels below accurately quantifiable concentrations, indicating almost complete inhibition in COX-2 activity (Supporting Information, Table 2). Strong COX-2 inhibition by indomethacin and indomethacin probe **7** was reported previously.^24^ Incubation with 10 μM negative control probe **8** resulted in slightly reduced PGE_2_ levels in the medium (Supporting Information, Table 2).

Similar to those of PGE_2_, medium IL-6 levels were reduced by DHEA in 1.0 μg mL^–1^ LPS-stimulated macrophages.^9^ After 24 h of incubation with 10 μM DHEA, a reduction of 41% in IL-6 medium levels (from 23.7 ± 8.9 to 14.0 ± 5.3 ng mL^–1^) was observed, not reaching significance. Incubations with 10 μM synthetic DHEA probes **2** and **3** did, however, significantly reduced IL-6 concentrations in the medium to 56% [10.5 ± 3.9 ng mL^–1^ (*P* < 0.01)] and 68% [7.6 ± 4.3 ng mL^–1^ (*P* < 0.01)], respectively. Statistical analysis using Tukey’s multiple comparison test indicated that incubation with 10 μM DHEA probe **3** was significantly more effective (*P* < 0.05) in reducing IL-6 production than that with 10 μM DHEA. Control probe **8** (Figure 2b), indomethacin **6**, and indomethacin probe **7** did not significantly affect IL-6 production (Supporting Information, Table 2). IL-6 production was also not significantly affected by AEA or AEA probe **5** when compared to that in vehicle incubation. Remarkably, incubation with 10 μM AEA probe **5** reduced IL-6 levels to 49% (12.2 ± 9.7 ng mL^–1^), whereas 10 μM AEA increased IL-6 levels to 122% (29.2 ± 10.9 ng mL^–1^), which corresponds to a significant (*P* < 0.05) difference between 10 μM AEA and 10 μM AEA probe **5** incubations using Tukey’s multiple comparison test (Supporting Information, Table 2). Although this outcome might suggest a distinct immunological effect of AEA and its corresponding probe **5** on IL-6, contradicting literature reports lead us to conclude that IL-6 is probably not a suitable marker for the immunological effects of AEA.^30−32^

In conclusion, we showed that the synthesized DHEA and indomethacin probes mimic the expected anti-inflammatory effects of the parent compounds in 1.0 μg mL^–1^ LPS-stimulated RAW264.7 macrophages, which was not the case for our negative control probe **8**.

### Synthetic Probes Localize around the ER and in Membrane Vesicles

**Figure 3:**
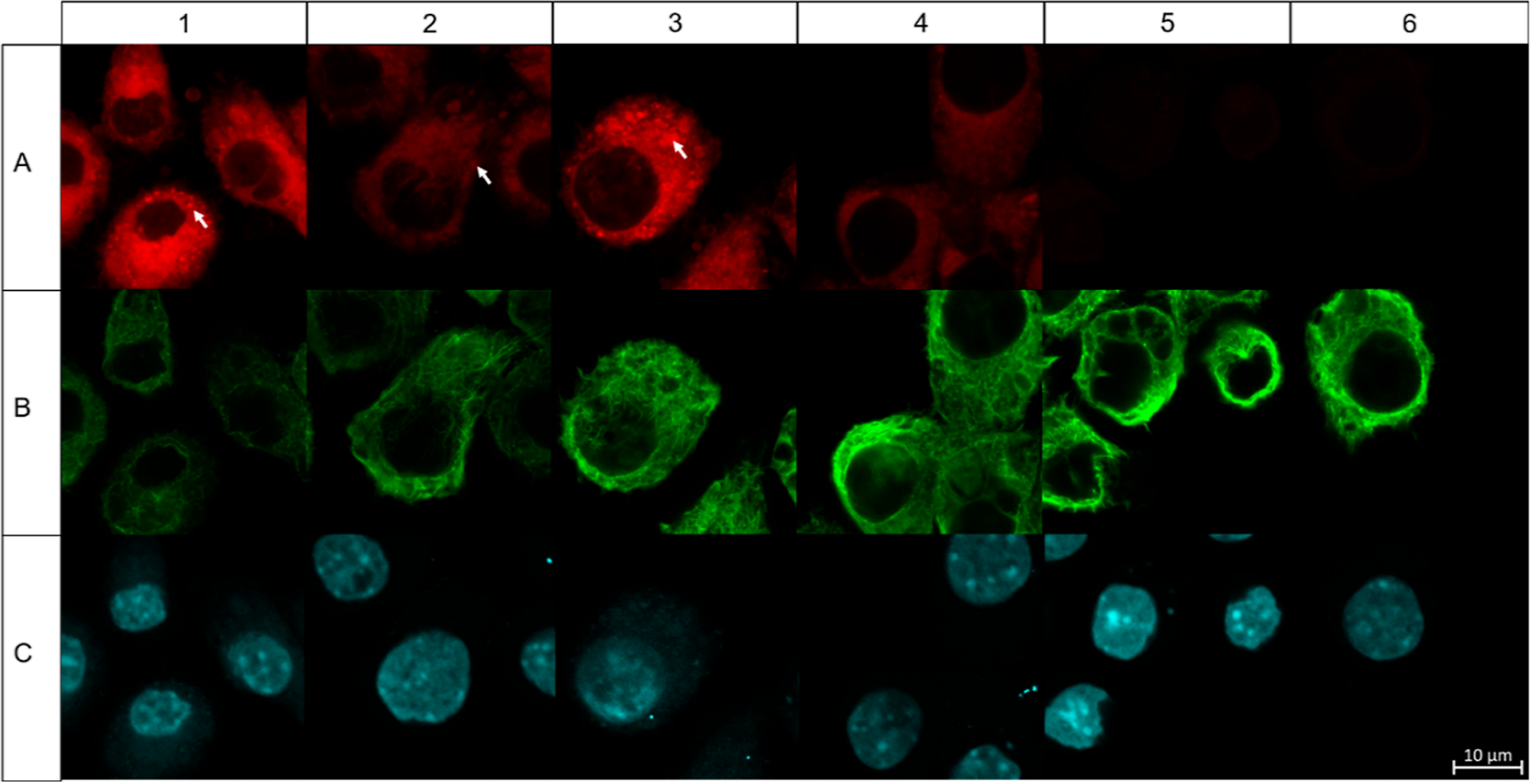
Confocal fluorescent images of 1.0 μg mL^–1^ LPS-stimulated RAW264.7 macrophages. Rows: (A) Lissamine rhodamine B channel after the CuAAC with lissamine rhodamine B. (B) Immunostained tubulin. (C) DAPI staining. Columns: (1) 10 μM DHEA probe **2**. (2) 10 μM DHEA probe **3**. (3) 10 μM AEA probe **5**. (4) 10 μM indomethacin probe **7**. (5) 10 μM probe **8**. (6) 0.1% EtOH vehicle. Arrows highlight vesicle compartmentalization. The scale bar applies to all images in the figure.

To better understand the biological functionality of our compounds, we first analyzed the in vitro localization of 4 h incubated 10 μM synthetic probes in 1.0 μg mL^–1^ LPS-stimulated RAW264.7 macrophages. The probes were visualized with lissamine rhodamine B PEG3 azide using tetrakis(acetonitrile)copper(I) hexafluorophosphate in chemically fixed cells that were immunostained with alpha-tubulin AlexaFluor488 and nuclear 4′,6-diamidino-2-phenylindole (DAPI) dsDNA staining (Figure 3). Control samples showed no nonspecific labeling of lissamine rhodamine B and AlexaFluor 488 (Supporting Information, Figures 3 and 4).

DHEA probe **2** was taken up by LPS-stimulated RAW264.7 macrophages in 4 h and localized around the nuclear periphery in cytosol, suggesting agglomeration in the endoplasmic reticulum (ER) and golgi system (Figure 3-1A). In addition, DHEA probe **2** clustered in spherical membrane domains. As the slightly different DHEA probe **3** showed similar intracellular localization to that of DHEA probe **2** (Figure 3-2A), we conclude that the observations relate to the localization of intact DHEA probes. Similarly, AEA probe **5** showed cytoplasmatic staining and an apparent high level of spherical domain compartmentalization (Figure 3-3A). Therefore, PUFA-derived amides tend to localize around the ER where they are generally catabolized by enzymes like COX-2.^33^ To show that the *N*-acylethanolamide probes are not extensively metabolized within cultured cells, additional metabolic tracing experiments using TLC fluorescence were performed (Supporting Information, Figure 5), applying a similar approach to that reported in the study by Thiele and co-workers.^34^ This experiment indicated that the parent DHEA probe 3 remained largely intact after incubation for 4 h with 1.0 μg/mL LPS-stimulated RAW264.7 macrophages, apart from a minor metabolite with slightly increased hydrophilicity as shown by a lower *R*_f_ value. Based on our previous observations, we hypothesize that this product most likely represents hydroxylated metabolites of the DHEA probe at the 13- and 16-position, which have been characterized as metabolites in this model.^17^ Together, these findings indicate that the intact probe remains available under the study conditions used. This is in line with previous results where we showed that intracellular DHEA concentrations in LPS-stimulated RAW264.7 macrophages remained stable for 48 h^17^ and that incubation with DHEA did not lead to measurable DHA levels in the medium of LPS-stimulated RAW264.7 macrophages.^9^

**Figure 4:**
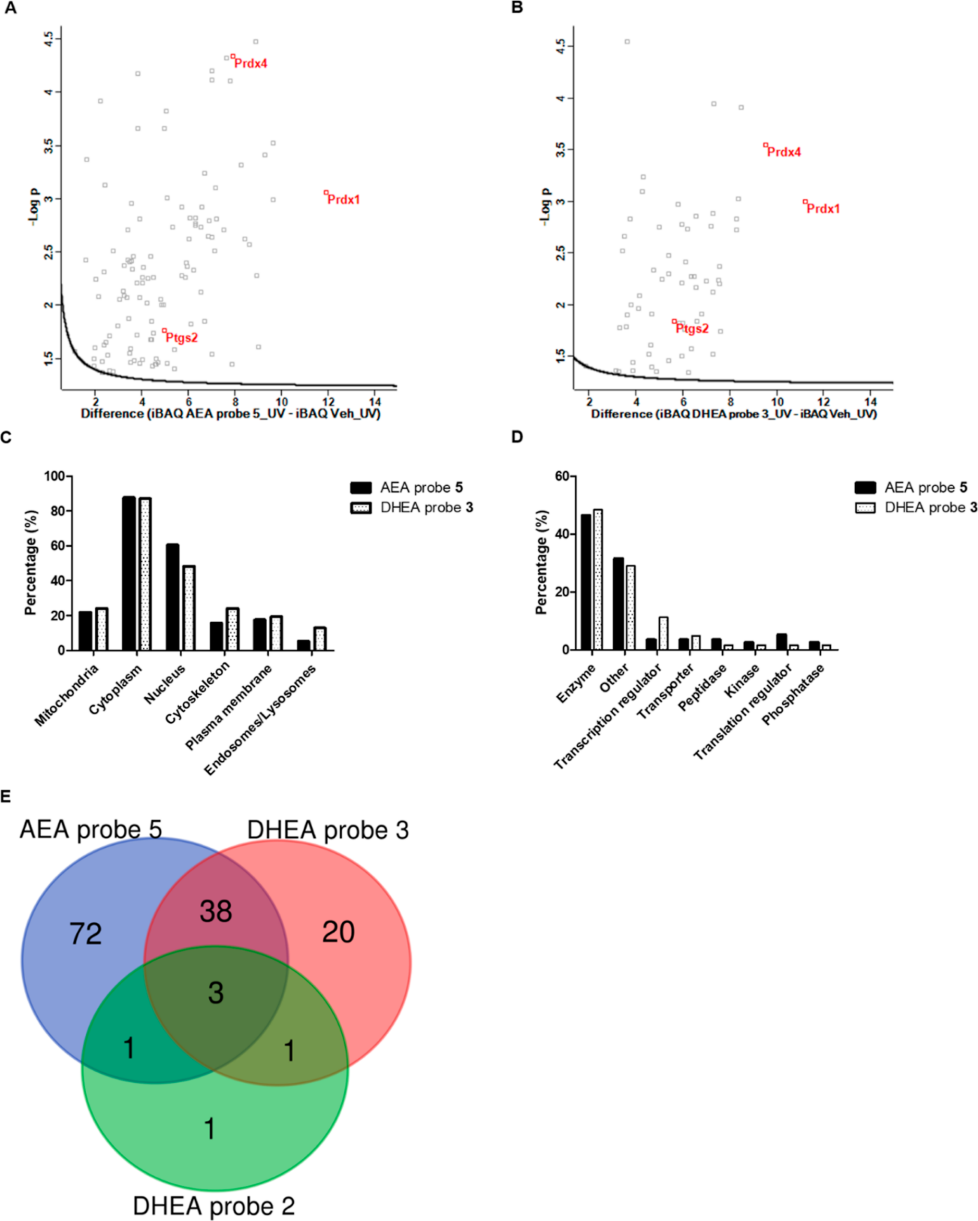
Proteomics analyses of DHEA probes **2** and **3** and AEA probe **5** (*N* = 3). (A) Volcano plot (FDR 0.05, S0 0.1) of AEA probe **5**. (B) Volcano plot (FDR 0.05, S0 0.1) of DHEA probe **3**. Both (A) and (B) show sequentially filtered data against the vehicle (0.1% EtOH control). *X*-axis represents 2log[iBAQ] value (probe–vehicle) differences, and *y*-axis represents −log *p* values. (C) Protein ontology of AEA probe **5** and DHEA probe **3** targets according to UniProt. (D) Cellular function of AEA probe **5** and DHEA probe **3** targets according to IPA. (E) Venn diagram of DHEA probes **2** and **3** and AEA probe **5** targets.

**Figure 5:**
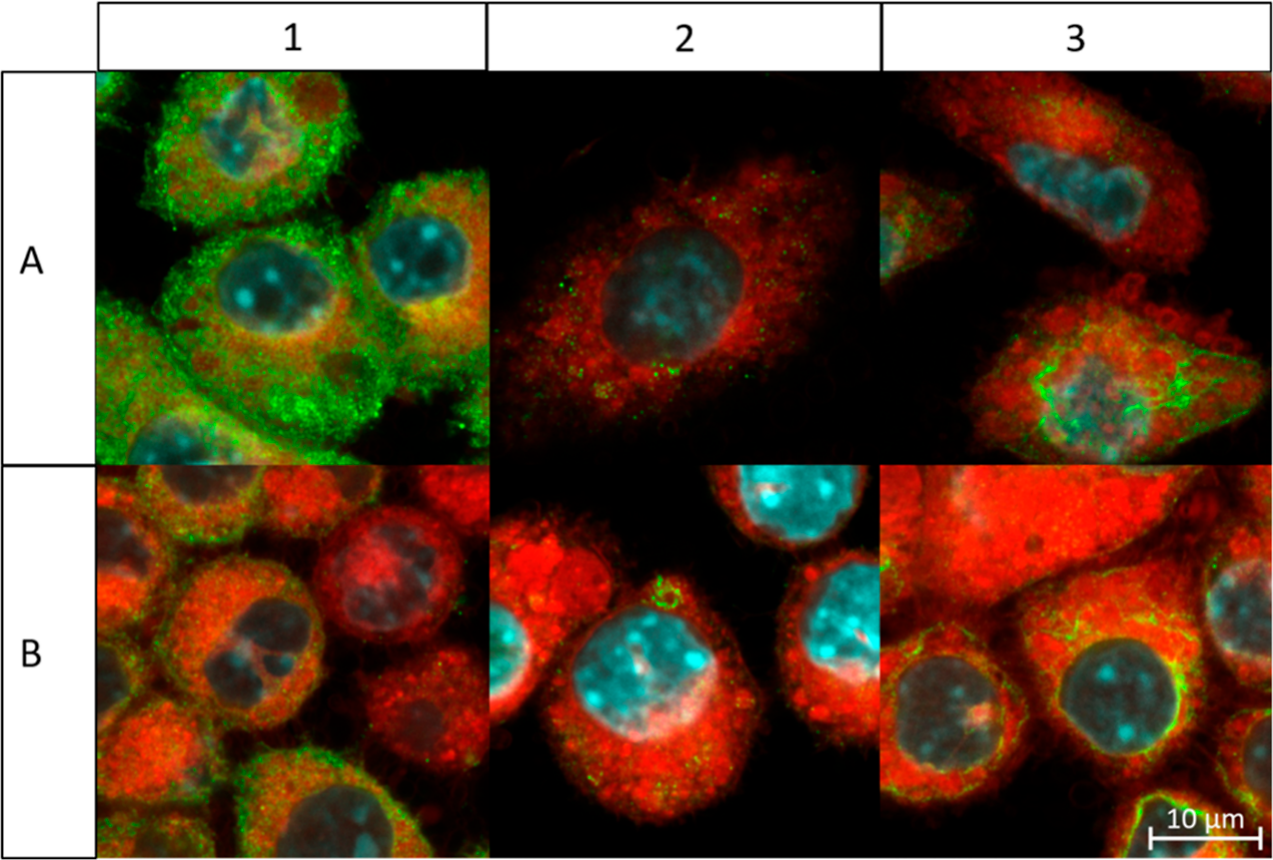
Confocal fluorescent images of probe-incubated RAW264.7 macrophages prestimulated for 4 h with 1.0 μg mL^–1^ LPS. (A) Cells incubated with fresh 1.0 μg mL^–1^ LPS and 10 μM DHEA probe **3** for an additional 4 h. (B) Cells incubated with fresh 1.0 μg mL^–1^ and 10 μM AEA probe **5** for an additional 4 h. Color overlays represent lissamine rhodamine B (red), AlexaFluor 488 antibody (green), and DAPI staining (blue). (1) Rac1 labeling, (2) Rab5c labeling, and (3) Ptgs2 labeling. The scale bar applies to all images in the figure.

Indomethacin probe **7** labeling was weaker than that with PUFA derivatives and was also localized inside the cytoplasm (Figure 3-4A). For control probe **8**, we observed almost no fluorescence labeling, suggesting its limited uptake or rapid breakdown (Figure 3-5A). Control incubations with the vehicle (0.1% EtOH) showed no background fluorescence from lissamine rhodamine B (Figure 3-6A).

The specific counterstaining of alpha-tubulin resulted in an AlexaFluor 488-stained cytoskeleton (Figure 3B), proving that fixation and immunofluorescence are successfully combined with click labeling of lipids, as reported previously.^26^ Z-stack projection of tubulin staining additionally showed fine tubulin structures indicating no or limited fixation damage during the preparation of the slides (Supporting Information, Figure 6 and Video S1). DAPI staining of nuclei clearly showed more intense labeling of condensed heterochromatic segments and less intense signals of euchromatic segments in the nucleus (Figure 3C). Our confocal fluorescence analysis provided evidence for the uptake of our PUFA probes **2** and **3** as well as indomethacin probe **7** in LPS-stimulated macrophages over a period of 4 h, resulting in localization around the ER and in membrane vesicles.

### Characterization of the PUFA-Derived Probe Interactome

Molecular interaction partners of our probes were identified by proteomic characterization of the interactome in 8 h LPS-stimulated and 4 h probe-incubated macrophages. Data analysis using iBAQ scores enabled identification of relative abundances of proteins.^35,36^ After filtering out nonspecific background interactions using signals from the vehicle (0.1% EtOH) incubations and statistical evaluation using a two-sample Student’s *t*-test (*p* < 0.05 and *t*-test difference > 1.0), 101 significantly enriched proteins were found for DHEA probe **2**, 198 proteins for DHEA probe **3**, 273 proteins for AEA probe **5**, and 55 proteins for indomethacin probe **7**. Sequential filtering using the randomly interacting control probe **8** and a second two-sample Student’s *t*-test (*p* < 0.05 and *t*-test difference > 1.0) resulted in 6 significantly enriched proteins for DHEA probe **2**, 62 proteins for DHEA probe **3**, 114 proteins for AEA probe **5**, and 4 proteins for indomethacin probe **7**. Significantly enriched proteins resulting from the sequential filtering were displayed against the vehicle (0.1% EtOH) treatment (Figure 4A,B and Supporting Information, Table 3). Despite sequential filtering, also with probe **8**, we identified targets such as ribosomal and cytoskeletal proteins, which are likely nonspecific, highly abundant protein targets that are often observed in similar affinity-based proteomic setups.^6,24,28^ Notwithstanding, comparison of enriched proteins with previous chemical proteomic enrichment studies revealed that 52 of the 114 AEA probe **5** targets were also enriched by A-DA and/or AEA-DA, two AEA-based chemical probes, in HEK293T/Neuro2a cells.^6^ Moreover, DHEA probe **3** and AEA probe **5** were found to interact with prostaglandin synthase 2 (Ptgs2; also known as COX-2); we and others previously demonstrated that this enzyme is involved in the oxygenation of AEA and DHEA (Figure 4A,B).^5,17^ These data support that our used methodology, comprising stringent washing of the affinity-labeled proteins with 3 × 3 washing steps, including a strong wash with 4 M urea and 0.4% SDS in ice cold 1× PBS containing a 1× cOmplete protease inhibitor, and sequential data filtering with a nonspecific binding control probe **8**, leads to detection of expected binding targets.

UniProt database and ingenuity pathway analysis (IPA) were used to identify cellular domain(s) and functional annotation of the protein targets (Figure 4C,D). Most proteins are localized in the cytoplasm (Figure 4C), confirming our observed cytoplasmatic localization (Figure 3). In addition to enzymes, we identified transcription and translation regulators, as well as transporters (Figure 4D). Comparison of the enriched proteins for DHEA probes **2** and **3** and AEA probe **5** revealed that only three proteins interacted with all three PUFA amide probes (Figure 4E). In total, 38 shared targets were found for DHEA probe **3** and AEA probe **5**, whereas 20 proteins were specific targets of DHEA probe **3**, and 72 proteins were specific targets of AEA probe **5**.

Both DHEA probe **3** and AEA probe **5** showed the strongest specific interaction with Prdx 1 (Prdx1) (Figure 4A,B and Table 1). Also, Prdx4 labeling was significant for both PUFA-derived amide probes (Figure 4A,B). Prdxs convert hydrogen peroxide to water and lipid hydroperoxides to alcohols, protecting the cells from ROS toxicity.^37,38^ Although many studies described the anti-inflammatory and protective effects of Prdxs, Prdx1 knockdown decreased inflammatory cytokine production and increased anti-inflammatory IL-10 production in LPS-stimulated RAW264.7 macrophages.^39^ The selective binding of our PUFA amides to Prdx1 could therefore be linked with the blockage of the Prdx1-induced inflammation in RAW264.7 cells. Previous proteomic screening with AA, the PUFA precursor of AEA, also showed an interaction of AA with ROS regulators in RAW264.7 macrophages, which was ascribed to the induction of lipid electrophile-driven coupling upon stimulation of the macrophages with the LPS mimetic Kdo_2_-lipid A.^29^ Although our methodology used diazirine crosslinking, lipid electrophile coupling to ROS scavengers could not be ruled out as a possible interaction mechanism between AEA, DHEA, and Prdxs. Interestingly, both DHEA and AEA were reported to induce the ROS production in HNSCC cells,^12,27^ and in 0.1 μg mL^–1^ LPS-stimulated mouse macrophages, 10 nM DHEA was found to reduce ROS production.^10^ In addition to Prdxs, we found ribosomal proteins, acetyl-CoA acetyltransferase (Acat1, mitochondrial), and the signaling regulator Rhoc in the top 10 AEA probe **5** interactors (Table 1).^40^

**Table 1:**
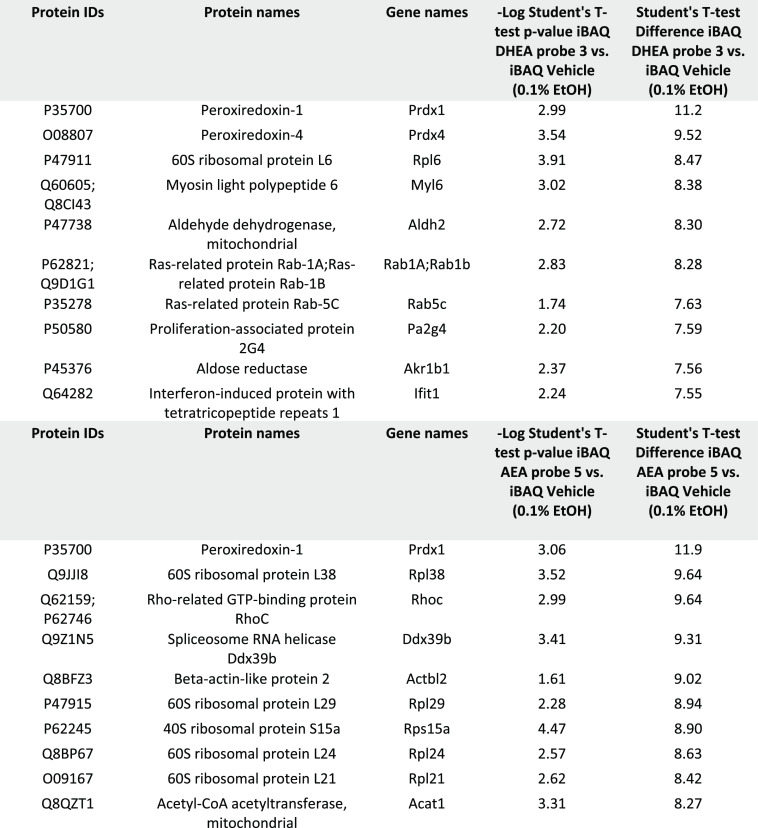
Protein Targets of DHEA Probe **3** (Top) and AEA Probe **5** (Bottom)^a^

a Top 10 protein differences between vehicle (0.1% EtOH) and 10 μM probes. Protein differences [2log[iBAQ] values (probe–vehicle)] and *p*-values were determined using a two-sample Student’s *t*-test (*p* < 0.05).

The top 10 DHEA probe **3** interactors showed two intracellular membrane trafficking proteins: Rab1a and Rab5c (Table 1). Rab1a regulates cell adhesion and cell migration via β1 integrin recycling and is localized in the ER and intracellular vesicle domains where DHEA was localized (Figure 3).^41^ Rab5c is a key regulator in early endosome trafficking and is involved in cell migration via β1 integrin recycling.^42^ Interestingly, these observations add to the ongoing debate concerning the uptake mechanism of PUFA derivatives, occurring via a currently unidentified membrane transporter or via passive diffusion.^8,43^ Characterization of endosomal proteins interacting with DHEA probe **3** and AEA probe **5** might indicate that lipid raft- and caveolae-dependent endocytosis is an uptake route for those PUFA-derivatives, as was previously suggested for AEA in the study by McFarland and co-workers.^44^ This hypothesis is further supported by the identification of the DHEA probe **3** interactor Dnm2, which has important functions in endosomal formation (Supporting Information, Table 3).^45^ Clearly, focused studies should be aimed at unraveling the uptake mechanisms of AEA and DHEA to support an endocytosis-dependent uptake mechanism. The transcription regulator Pa2g4 and the pro-inflammatory immune regulator Ifit1 were also significantly bound by DHEA probe **3**. Recently, we reported that the expression of Ifit1 was reduced as a result of incubation with 5 μM 13-HDHEA and 16-HDHEA (hydroxydocosahexaenoyl ethanolamide), products of the interaction between DHEA and COX-2.^18^ As a significant Ifit1 interaction with AEA probe **5** was not observed here, it appears that Ifit1 is only involved in the signaling of DHEA or its metabolites. Interestingly, previously described endocannabinoid-interacting proteins, for example, CB1, CB2, and GPR55, as well as TRPV1 and PPAR,^1,3,4,7,8,19,21^ are not identified in our model, likely due to poor expression of those proteins in LPS-stimulated RAW264.7 macrophages.^9^

In conclusion, we observed Prdx-1, Prdx-4, Rhoc, and Acat1 as important AEA probe interactors in LPS-stimulated RAW264.7 macrophages. From these targets, only Prdx-1 was already characterized as a potential AEA target.^6^ In addition to these AEA targets, Prdx1, Rab1a, Rab5c, Pa2g4, and Ifit1 were identified as most important DHEA-interacting proteins. Our chemical biological high-throughput method enabled the identification of novel PUFA-amide targets that could not have been revealed with classical endocannabinoid receptor binding studies.

### IPA Indicates the Involvement of GTPase Signaling

Functional IPA revealed that enriched protein targets of DHEA probe **3** and AEA probe **5** are mainly involved in Rho family GTPase signaling and actin regulation (Supporting Information, Table 4). A notable protein in the regulation of this pathway is Rac1, which was identified as a significant interactor with DHEA probe **3** and AEA probe **5** but did suffer from relatively weak spectral matching scores and should, therefore, be interpreted with care. Notwithstanding, 21 of the 62 protein hits from DHEA probe **3** and 48 of the 114 protein hits from AEA probe **5** (including Rac1 itself) were identified as (putative) Rac1-interacting proteins to further support the potential role of Rac1 in PUFA-derived amide-mediated GTPase signaling.^46,47^ Rho GTPase signaling plays an important role in ROS signaling, cell migration, cytoskeletal remodeling, and actin regulation.^47−49^ Indeed, these phenotypic effects can be related to previously reported functions of DHEA and AEA. The effects of DHEA and AEA on ROS regulation were already described above, and other studies have reported antimigratory properties of AEA^3,4,10^ and DHEA,^10^ including its oxidized metabolites.^15,16,18^ In addition to small GTPase signaling, several actin and myosin-related proteins were also significantly affected by DHEA probe **3** and AEA probe **5**, suggesting that cytoskeletal remodeling is a prerequisite for migration.

Disease and function analysis in IPA showed indications of a response similar to that for viral infections for DHEA probe **3** proteins and cellular organization, response to viral infections, and reduced cell death for AEA probe **5** proteins. However, as IPA uses experimental results from literature reports, it is suboptimal for our current methodology, which was aimed at unraveling novel interactions. In conclusion, links to ROS signaling, actin remodeling, and cell migration were obtained using IPA, but additional research is required to prove that these effects are mediated by GTPase signaling via DHEA and AEA interactions.

### Colocalization Supports DHEA and AEA Interactions with Rac1 and Ptgs2

To support a suggested role in endosomal trafficking, Rho GTPase signaling, and lipid metabolism, we performed fluorescence confocal imaging studies staining DHEA probe **3** and AEA probe **5** with lissamine rhodamine B and Rab5c, Rac1, and Ptgs2 with AlexaFluor488 immunostaining (Figure 5). RAW264.7 macrophages were prestimulated for 4 h with 1.0 μg mL^–1^ LPS and subsequently exposed to 10 μM DHEA probe **3** or 10 μM AEA probe **5** and 1.0 μg mL^–1^ LPS for 4 h, mimicking conditions of the proteomic experiment to allow correlations. In addition to midplane images (Figure 5), Z-stack series were recorded, showing that midplane images are representative for the fluorescence localization (Videos S2, S3, S4, S5, S6, and S7). Cytoplasmatic localization was observed for the green Rac1 channel that often colocalized with the spectrally well-separated red PUFA-derived probes in the cell (Figure 5-1A,B), indicating the potential interaction of Rac1 with the PUFA probes. Nonetheless, areas that contained only a signal corresponding to Rac1 or to the PUFA-derived probes were also obtained. Rab5c immunostaining resulted in localization of small vesicles, possibly representing late endosomes, but showed no clear colocalization with our probes (Figure 5-2A,B). Ptgs2 staining showed main Ptgs2 signals at the cytoplasmic face of the nuclear periphery, known to be rich in the ER where Ptgs2 synthesis takes place (Figure 5-3A,B).^50^ Interestingly, the fluorescence signal of the PUFA amide probes was also relatively strong in the nuclear periphery. Although areas of colocalizing Ptgs2- and PUFA-derived amide probes were observed, the signal from Ptgs2 and the probes did not fully colocalize. Even though colocalization within the same voxel (∼270 × 270 × 1000 nm) does not directly prove a molecular interaction, this is a prerequisite. Nevertheless, our observations further support that PUFA-derived amides could interact with multiple partners including Rac1 and Ptgs2. Direct proof for the interaction between the PUFA amides and Ptgs2 was indeed previously demonstrated by the Ptgs2-mediated catabolic conversion of AEA and DHEA.^1,5,17^ Controls without primary Rac1, Rab5c, and Ptgs2 antibodies showed no immunofluorescence, indicating specificity of the antibodies and immunostaining protocol (Supporting Information, Figure 6). In conclusion, confocal fluorescence microscopy showed colocalization between the PUFA-derived probes and Rac1 and Ptgs2 as a prerequisite for a molecular interaction. Together with the previously characterized biochemical interaction in the proteomic setup and the reported metabolic interaction between the PUFA amides and Ptgs2, these data strongly support a functional interaction.

## Conclusions

Chemical probes of DHEA and AEA containing two specific tagging functionalities showed that PUFA amides interact with Prdx1, Prdx4, endosomal proteins such as Rab1 and Rab5c, and proteins of the GTPase-signaling pathway in LPS-stimulated RAW264.7 macrophages, rather than only with Ptgs2. In addition, fluorescence labeling of our probes indicated localization in the cytosol, and seemingly the ER and Golgi system, next to compartmentalization in membrane vesicles. Colocalization experiments using confocal fluorescence microscopy supported interactions between PUFA-derived probes and the small GTPase-regulating protein Rac1 as well as Ptgs2. Together, these observations provide novel insights into cellular PUFA amide interactions and their effects on cytoskeletal remodeling, cell migration, and ROS regulation. In addition, our results provide evidence for passive endosomal uptake mediated by lipid rafts.

In view of the unnaturally high lipid probe concentrations used in our and similar in vitro studies, future research should strengthen the interactome data sets by further research on the biological relevance of the identified proteins targets. Additional details regarding PUFA amide–protein interactions that regulate ROS formation, cytoskeletal remodeling, and cell migration may be obtained, as well as support of a lipid raft-dependent uptake mechanism of PUFA-derived amides. This might enable translation of the in vitro effects of DHEA and AEA to in vivo models and ultimately to human metabolism.

## Supporting Information Available

Summary of all significant protein hits per probe (XLSX)
Overview of the IPA pathways involved for the probe-targeting proteins (XLSX)
Z-stack of the confocal fluorescent images of RAW264.7 macrophages stained with Indomethacin probe 7 and Tubulin (AVI)
Z-stack of the confocal fluorescent images of RAW264.7 macrophages stained with DHEA probe 3 and Rac1 (AVI)
Z-stack of the confocal fluorescent images of RAW264.7 macrophages stained with DHEA probe 3 and Rab5c (AVI)
Z-stack of the confocal fluorescent images of RAW264.7 macrophages stained with DHEA probe 3 and Ptgs2 (AVI)
Z-stack of the confocal fluorescent images of RAW264.7 macrophages stained with AEA probe 5 and Rac1 (AVI)
Z-stack of the confocal fluorescent images of RAW264.7 macrophages stained with AEA probe 5 and Rab5c (AVI)
Z-stack of the confocal fluorescent images of RAW264.7 macrophages stained with AEA probe 5 and Ptgs2 (AVI)
Cytotoxicity, prostaglandin, and cytokine release in probe-incubated RAW264.7 macrophages; control experiments for the confocal fluorescence microscopy; fluorescent TLC experiment to prove the cellular stability of DHEA probe 3; and extended methodology section including synthesis, purification, characterization, and quantification of the chemical PUFA-derived amide probes (PDF)

## Supplementary Material

cb1c00355_si_002.xlsx

cb1c00355_si_003.avi

cb1c00355_si_004.avi

cb1c00355_si_005.avi

cb1c00355_si_006.avi

cb1c00355_si_007.avi

cb1c00355_si_008.avi

cb1c00355_si_009.avi
