# Supplementary material for: PUFA-Derived N-Acylethanolamide Probes Identify Peroxiredoxins and Small GTPases as Molecular Targets in LPS-Stimulated RAW264.7 Macrophages
Source: ACS Chem Biol. 2022 Jul 22;17(8):2054–64. doi: 10.1021/acschembio.1c00355 (PMC9396616; doi:10.1021/acschembio.1c00355)
Supplement: Supplementary file 10 — cb1c00355_si_010.pdf [file cb1c00355_si_010.pdf]

Supporting information

For

PUFA-derived *N*-acylethanolamide probes identify peroxiredoxins and small GTPases as molecular targets in LPS-stimulated RAW264.7 macrophages

Ian-Arris de Bus[1,2], Antoine H.P. America[3], Norbert de Ruijter[4], Milena Lam[2], Jasper W. van de Sande[2], Mieke Poland[1], Renger F. Witkamp[1], Han Zuilhof[2,5,6], Michiel G.J. Balvers[1†*], Bauke Albada[2†*]

1) Division of Human Nutrition and Health, Wageningen University & Research, Stippeneng 4, 6708 WE, Wageningen, The Netherlands
2) Laboratory of Organic Chemistry, Wageningen University & Research, Stippeneng 4, 6708 WE, Wageningen, The Netherlands
3) Wageningen Plant Research, Business Unit Bioscience, Wageningen University & Research, Droevendaalsesteeg 1, 6708 PB Wageningen, The Netherlands
4) Laboratory of Cell Biology, Wageningen University & Research, Droevendaalsesteeg 1, 6708 PB Wageningen, The Netherlands
5) School of Pharmaceutical Sciences and Technology, Tianjin University, 92 Weijin Road, Tianjin 300072, People's Republic of China.
6) Department of Chemical and Materials Engineering, Faculty of Engineering, King Abdulaziz University, 21589 Jeddah, Saudi Arabia.

[†]Both authors contributed equally to this manuscript
*Corresponding Authors:

Bauke Albada
Laboratory of Organic Chemistry,
Wageningen University & Research
Stippeneng 4, 6708 WE,
Wageningen, The Netherlands
bauke.albada@wur.nl

Michiel Balvers
Division of Human Nutrition and Health
Wageningen University & Research
Stippeneng 4, 6708 WE,
Wageningen, The Netherlands
michiel.balvers@wur.nl

**Extended Methods section**	**3-6**
Materials	3
Cell culture	3
Cytotoxicity assay	4
Prostaglandin E_2 ELISA	5
IL-6 ELISA	5
Proteomic workup	5
Proteomic LC-MS analysis	6
Proteomic data analysis	6
Immunostaining and click fluorescence protocol	7
Fluorescent microscopy	7
Fluorescent metabolic tracing TLC	7
**Supplemental figures**	**9-12**
Supplementary Figure 1	9
Supplementary Figure 2	9
Supplementary Figure 3	10
Supplementary Figure 4	10
Supplementary Figure 5	11
Supplementary Figure 6	11
Supplementary Figure 7	12
**Supplemental tables**	**13-14**
Supplementary Table 1	13
Supplementary Table 2	14
**Synthesis of Chemical probes**	**15-20**
**Materials**	**15**
**Synthetic Methodology**	**15-20**
tert-butyl 3-oxobutylcarbamate (c)	15
tert-butyl (2-(3-methyl-3H-diazirin-3-yl)ethyl)carbamate (e)	16
diazirine amine (f)	17
DHEA probe **3**	17
DHEA probe **2**	18
AEA probe **5**	18
Indomethacin probe **7**	19
Control probe **8**	19
**Purification and quantification of probes**	**20**
Preparative HPLC purification of DHEA probe **2** and AEA probe **5**	20
Semi-Preparative HPLC purification of DHEA probe **3**	20
Preparative HPLC purification of Control probe **8**	20
NMR Quantification of DHEA probe **3**	20
**NMR spectra**	**21**
^{1}H-NMR DHEA probe 3	21
COSY NMR DHEA probe 3	22
HSQC NMR DHEA probe 3	23
TOCSY NMR DHEA probe 3	24
^{1}H-NMR DHEA probe 2	25
^{13}C-NMR DHEA probe 2	26
COSY NMR DHEA probe 2	27
HSQC NMR DHEA probe 2	28

^{1}H-NMR AEA probe 5	29
^{13}C-NMR AEA probe 5	30
COSY NMR AEA probe 5	31
HSQC NMR AEA probe 5	32
^{1}H-NMR Indomethacin probe 7	33
^{13}C-NMR Indomethacin probe 7	34
COSY NMR Indomethacin probe 7	35
HSQC NMR Indomethacin probe 7	36
^{1}H-NMR Control probe 8	37
^{13}C-NMR Control probe 8	38
COSY NMR Control probe 8	39
HSQC NMR Control probe 8	40
**Calibration Curve and NMR spectra for DHEA probe 3 quantification**	**41-42**
^{1}H NMR of 3.8 mM 1,2,4,5-Tetrachloro-3-nitrobenzene	41
^{1}H NMR of the quantification run of DHEA probe **3**	42
Calibration curve of 1,2,4,5-Tetrachloro-3-nitrobenzene	42
**References**	**43**

Extended methods section

Materials

DHEA (≥ 98%) was purchased from Cayman Chemical and supplied by Sanbio B.V. AEA (100 % purity from HPLC) was obtained from Tocris Chemicals. Ammonium bicarbonate (≥99%), azide-PEG3-biotin conjugate, dimethyl sulfoxide (DMSO) (BioUltra, for molecular biology, ≥99.5%), lipopolysaccharides (LPS) from *E. coli* O111:B4 (L3024) , saponin (from *Quillaja bark*), (+)-sodium L-ascorbate (≥99%, BioXtra), tris(3-hydroxypropyltriazolylmethyl)amine (THPTA) (≥95%), triton-X100, tween® 20 (for molecular biology), and tetrakis(acetonitrile)copper(I) hexafluorophosphate (≥97%) were purchased from Sigma Aldrich. Copper(II)sulfate pentahydrate (≥99%, for analysis), D,L-1,4-dithiothreitol (DTT) (≥99%, for biochemistry), indomethacin (≥98%), iodoacetamide (IAA) (≥98%), Pierce™ BCA protein assay kit, protein LowBind tubes, Pierce™ C18 tips, Pierce™ streptavidin magnetic beads were purchased from Fisher Scientific. Ethylenediaminetetraacetic acid (EDTA) disodium salt dihydrate, Pharmpur® was obtained from Scharlab. Trypsin Gold (Mass Spectrometry Grade) was obtained from Promega Benelux B.V. Formic acid (≥99%, ULC/MS grade), trifluoroacetic acid (≥99.95%, HPLC grade) were obtained from Biosolve B.V. 1x PBS (pH 7.4), Dulbecco's Modified Eagle's Medium (DMEM), and penicillin and streptomycin were purchased from Corning. Fetal calf serum (FCS) was obtained from Biowest and supplied by VWR International B.V. Methanol (≥99.9%, HiPerSolv CHROMANORM®, ULTRA for LC-MS, suitable for UPLC/UHPLC-MS instruments), Acetonitrile (ACN) (≥99.9%, HiPerSolv CHROMANORM® for LC-MS, suitable for UPLC/UHPLC instruments), HEPES-2-[4-(2-Hydroxyethyl)-1-piperazinyl]-ethane sulfonic acid free acid, high purity (≥99%), and sodium dodecyl sulfate (SDS) (≥99%), biotechnology grade were obtained from VWR International B.V. Urea (≥99%, Molecular biology grade) was purchased from SERVA Electrophoresis GmbH. 1x cOmplete™ protease inhibitor, and LDH cytotoxicity kit were purchased from Roche. Ammonium acetate, and gelatin powder EMPROVE® (≥99%) were obtained from Merck. Ethanol (absolute for analysis EMSURE®), and Probumin® BSA were purchased from Merck. Lissamine rhodamine B PEG3 azide (≥95%) was purchased from Tenova Pharma. Anti-alpha tubulin antibody - microtubule marker (rabbit polyclonal ab18251), recombinant anti-COX2/cyclooxygenase 2 antibody [EPR18376-119] - C-terminal (rabbit monoclonal ab188184), goat anti-rabbit IgG H&L (Alexa Fluor® 488) (ab150077) were purchased from Abcam (Cambridge, UK). RAC1 rabbit polyclonal antibody (PA1-091), RAB5C rabbit polyclonal antibody (PA5-39408), and Invitrogen™ DAPI (4',6-Diamidino-2-Phenylindole, Dihydrochloride) were obtained from Invitrogen. PBS citifluor (AF3), and glycerol citifluor (AF4) were purchased from Citifluor. Ultrapure water was filtered by a MilliQ integral 3 system from Millipore.

Cell culture

All cell experiments were performed in RAW264.7 macrophages (American Type Culture Collection) cultured in DMEM containing 10% FCS and 1% penicillin and streptomycin (P/S). Cells were incubated at 37 °C and 5% CO_2 in a humidified incubator.

Cytotoxicity and anti-inflammatory effects

Macrophages were seeded at $2.5 \cdot 10^5$ cells mL^{-1} and incubated overnight in 24-wells plates (Corning Life Sciences), containing 0.5 mL medium per well. Medium of adherent cells was discarded and replaced with fresh medium containing 5 or 10 µM of the compounds (in 0.1% v/v EtOH for PUFA conjugates, in 0.1%

v/v DMSO for indomethacin) or a vehicle (0.1% v/v EtOH or 0.1% v/v DMSO) control. Cells were pre-incubated with the compounds for 30 min before stimulation with 1.0 µg mL^{-1} LPS in 0.1% PBS or 0.1% PBS control. After LPS addition, cells were incubated for 24 h in the dark (aluminum foil covered) to protect the probe from incidental UV exposure. Finally, medium was collected and IL-6, PGE_2 and LDH concentrations were quantified.

Proteomic experiment

In 100 mm culture dishes, RAW264.7 macrophages were seeded at a density of (0.5–1.0)•10^6 cells mL^{-1} in 15 mL medium. After overnight culture, cells were stimulated with 5 mL fresh medium containing 1.0 µg mL^{-1} LPS in 0.1% PBS. After 4 h of LPS stimulation, cells were incubated with 5 mL of fresh medium containing 1.0 µg mL^{-1} LPS in 0.1% PBS and 10 µM of the synthetic probes in 0.1% EtOH or DMSO. Probe-treated macrophages were incubated for 4 h in the dark (aluminum foil covered). Following incubation, medium and non-adherent cells were removed, after which samples were placed on ice. Illumination with UV light was performed during 10 min at 366 nm and 1 mJ cm^{-2} with an UVP-C1000 crosslinker equipped with five 8W light bulbs (Supplementary Figure 1), or under normal lamp light as control. After light treatment, cells were collected by scraping in 5 mL ice cold 1x PBS and used in the proteomic workup.

Fluorescence and immunostaining

Immunostaining and additional fluorescent click labelling were based on an existing protocol from Gaebler *et al.*[1] RAW264.7 macrophages were seeded in Ibidi µ-Slide 8 Well ibiTreated polymer coverslips (Ibidi GmbH) with a density of 2.5•10^5 cells mL^{-1}, containing 300 µL cell suspension per well. Cells were allowed to grow overnight and then pre-stimulated for 4 h with 1.0 µg mL^{-1} LPS, prior to a 4 h incubation with fresh medium containing 10 µM probe or 0.1% EtOH (vehicle) and 1.0 µg mL^{-1} LPS (LPS pre-stimulation). Alternatively, cells were directly incubated with 10 µM probes or vehicle together with 1.0 µg mL^{-1} LPS for 4 h (no LPS pre-stimulation). After incubation, medium of adherent cells was discarded and cells were irradiated at 366 nm for 5 min on ice (lamp conditions in *probe incubation*), or placed on ice under 'control' (normal lamp light) conditions. Immunostaining and fluorescent click labelling was performed as described in the relevant method sections.

Cytotoxicity assay

To evaluate the cytotoxicity of the added compounds and probes, an LDH cytotoxicity Kit (Roche) was used to measure LDH leakage. Extracellular LDH was determined by adding 100 µL of a reagent solution (1:45 (v/v) of LDH reagent 1:LDH reagent 2) to 100 µL of sample medium. Plates were incubated for 30 min followed by quenching the reaction with 50 µL of 1.0 M HCl. The absorbance was read using a plate reader at 492 nm. As a control for maximum cytotoxicity, cells were incubated with 1% Triton-X100.

Prostaglandin E_2 ELISA

Medium concentrations of PGE_2 in incubated medium samples were assessed with a prostaglandin E_2 ELISA Kit – Monoclonal (Cayman chemical). The ELISA was performed according to the recommendations of the manufacturer on 5 or 50x diluted medium samples.

IL-6 ELISA

To assess medium concentrations of IL-6 in incubated RAW264.7 macrophages, a mouse IL-6 DuoSet ELISA from R&D systems was used. The ELISA was performed according to the recommendations of the manufacturer on 100x diluted medium samples.

Proteomic workup

Scraped cells in 5 mL ice cold 1x PBS were centrifuged at 3180 rcf for 5 min at 4 °C. Then cells were washed with 1 mL of ice cold 1x PBS containing 1x cOmplete™ protease inhibitor, followed by centrifugation at 3180 rcf for 5 min at 4°C. The cell pellet was washed with ice cold 1 mL, and 0.5 mL 1x PBS containing 1x cOmplete™ protease inhibitor, before sonicating for 3 x 10 s at 10% amplitude with a Branson Digital Sonifier 450 cell disruptor (Branson Ultrasonics). The sonicated suspension was transferred to low protein binding tubes and centrifuged at 16000 rcf for 20 min at 4 °C. The protein containing supernatant was stored at -20 °C, and the protein concentration was determined using a BCA assay.

Proteomes were diluted to 500 µL of 2 mg mL^{-1} protein and allowed to react for 1 h at RT with 10 µL THPTA (100 mM in H_2O), 10 µL $CuSO_4$ pentahydrate (20 mM in H_2O), 10 µL sodium L-ascorbate (300 mM in H_2O), and 10 µL azide PEG3 Biotin (5 mM in DMSO). After this click reaction, 1 mL of ice cold MeOH was added to precipitate the proteins at -80 °C for 1 h. The precipitated proteins were centrifuged at 15000 rcf for 10 min, dried in the air for 15 min, and subsequently dissolved in 500 µL 1x PBS containing 1x cOmplete™ protease inhibitor, and 0.4% SDS. Subsequently, the biotinylated proteins were incubated overnight with 50 µL of Pierce™ Streptavidin Magnetic Beads at 4 °C in an end-over-end shaker. Next day, the beads were washed three times with 250 µL wash buffer 1 (ice cold 1x PBS containing 1x cOmplete™ protease inhibitor), three times with 250 µL wash buffer 2 (4M Urea, 0.4% SDS in ice cold 1x PBS containing 1x cOmplete™ protease inhibitor), and finally again three times with 250 µL wash buffer 1. Washed beads were suspended in 25 µL 8M Urea, followed by 75 µL 50 mM ammonium bicarbonate. Then 10 µL of 50 mM DTT was added to achieve cleavage of disulfide bridges during 30 min at 37 °C. After cooling bead suspensions, 10 µL of 100 mM IAA was added to alkylate the free thiols. Alkylation was followed by addition of 1 µL of 0.5 µg μL^{-1} trypsin, and samples were subsequently incubated for 16 h at 37 °C to digest the proteins. After trypsinization, the peptide solution was transferred to new low protein binding tubes while magnetically removing the Pierce™ Streptavidin Magnetic Beads. To the resulting peptide solution 6 µL of 10% TFA was added. Peptides were cleaned using Pierce™ C18 Tips according to the description of the manufacturer and eluted using 100 µL of 50% ACN and 0.1% FA in ultrapure water. The purified samples were evaporated in a Speedyvac concentrator at 30 °C.

Proteomic LC-MS analysis

Dried peptides were dissolved in 20 µL 2% ACN in 0.1% FA and 5 µL was injected on a nanoLC-MS system (EASY-LCII connected to Q-exactive^{PLUS}, ThermoScientific,). Peptides were trapped on a 2 cm x 0.1 mm C18

trap column, and separated on an 8 cm x 0.75 mm C18 analytical column (PepSep) using a flow rate of 300 nL min^{-1}. Sample loading and trapping was performed in buffer A (0.1% FA in ultrapure water), and elution was performed with a 20 min gradient going from 2% to 30% buffer B (0.1% FA in ACN), followed by column regeneration at 80% buffer B and re-equilibration at 2 % buffer B. The nanoLC eluate was directly sprayed into the source of the Q-exactive by Flex-ion nanospray, using a PepSep nanospray needle at 2.3 kV ESI potential. MS acquisition was performed using a DDA method with alternating MS1 scan at resolution 70000 profile mode, AGC target $3•10^6$, maxIT 50 ms, scan range 500–1400 *m/z*, and subsequently 8 MS2 scans centroid mode, resolution 17500, AGC target $5•10^4$, maxIT 100 ms, with isolation window 1.6 *m*/*z* at NCE=28 with preferred peptide match ions of charges 2, 3 or 4 and a dynamic exclusion window of 30 s.

Proteomic data analysis

Proteomic LC-MS data analysis from triplicate incubations was performed using Maxquant software version 1.6.17.0 matching to the Uniprot reference protein database of *Mus musculus* (taxon 10090) from October 2019. Annotation was performed using peptide spectral matching (PSM) with FDR 0.01, and protein FDR 0.01. Intensity-based absolute quantification (iBAQ) scores were calculated for each annotated protein, allowing match between runs. The iBAQ score is defined as the sum of peak intensities of all peptides matching with a specific protein, divided by the number of theoretically observable peptides of that protein (based on trypsin cleavage sites).[2] Data filtering was performed using Perseus software version 1.6.13.0. Only annotated proteins with MS/MS counts (=number of MS/MS spectra of a protein that are matched with a peptide of that protein) >2 were taken into account. All iBAQ scores were log2 transformed, and only proteins with annotations in all replicates of at least one group were taken into account. All missing values were randomly replaced with normally distributed values containing a downshift of 2 and a width of 0.2. Filtering of the data was performed, continuing only with significant proteins ($P<0.05$) after a two-sample Student's t-test against the UV-treated vehicle (0.1% EtOH). Subsequently, filtering against the UV-treated control probe **8** was performed using a second two sample Student's t-test, continuing only with proteins that significantly ($P<0.05$) interacted with the PUFA-derived and indomethacin probes. For analytical purpose the resulting dataset was compared to that of the UV-vehicle (0.1% EtOH) in all data analyzes. The significantly interacting proteins observed after sequential filtering were analyzed using Uniprot Knowledgebase (https://www.uniprot.org/) and using Ingenuity Pathway Analysis (IPA®) (http://www.ingenuity.com/science/knowledgebase) from Qiagen Benelux B.V. (Venlo, NL) in December 2020. The Uniprot Knowledgebase is a free online database containing sequences and annotations extracted from literature.[3] The Ingenuity Knowledge Base is a knowledge repository that houses biological and chemical relationships extracted from the scientific literature. Using IPA, interconnected pathways, protein interactions, and clustering of protein functionality and cellular localization was performed for the extracted proteins.

Immunostaining and click fluorescence protocol

Immunostaining and click fluorescence were performed according to a previously described method by Gaebler *et al.*[1] The cells were washed 3 x 300 µL 1x PBS, and fixed in 300 µL 4% paraformaldehyde in PBS for 10 min at room temperature. After 10 min fixation, cells were washed with 3 x 300 µL 1x PBS. Cells were permeabilized for 15 min. in permeabilization buffer consisting of 1x PBS/1% gelatin/0.01% saponin.

This was followed by overnight incubation with the primary antibody in PBS/1% gelatin/0.01% saponin at 4 °C, or in PBS/1% gelatin/0.01% saponin without the primary antibody at 4 °C for the control labelling. The next day, the cells were washed three times with 300 µL PBS, applying 5 min per wash step. The samples were then treated with the (GaR-IgG-Alexa Fluor® 488) secondary antibody in PBS/1% gelatin/0.01% saponin for 1 h in the dark, after which the samples were again washed with 3 x 300 µL 1x PBS.

The immunostaining protocol was followed by a lissamine rhodamine B staining protocol using Cu(I)-mediated azide alkyne click reaction. The samples were pre-washed with 300 µL 100 mM HEPES/KOH pH 7.4 (click buffer). Then 300 µL of click buffer containing 13 µM lissamine rhodamine B PEG3 azide was added, to which 6 µL of 100 mM CuTFP in acetonitrile was added or 6 µL of acetonitrile for the control incubations. The reaction was allowed to stand for 1 h at RT in the dark. Hereafter, the samples were washed with 300 µL click buffer, 20 mM EDTA solution, 155 mM ammonium acetate, and 1x PBS. Samples were washed again in 300 µL citifluor PBS and thin mounted in citifluor glycerol containing 1.43 µM DAPI. Next, they were stored at 4 °C covered in aluminum foil, before analyzing with a fluorescent microscope.

Fluorescent microscopy

Microscopy was performed using a Zeiss LSM 510-META Confocal laser scanning Zeiss microscope, equipped with a Plan-Apochromat 63x/NA1.4 oil DIC lens. Representative images were recorded using 2 individual tracks for 1 sample: In the first track DAPI was excited with a 405 nm laser line and emission recorded at wavelength 420-490 nm; and lissamine rhodamine B was excited with a 543 nm laser line and emission recorded at wavelength above 560 nm using a long pass filter. In the second track AlexaFluor488 was excited using 488 nm laser line and emission recorded between 505-570 nm. Cells were focused at the midplane and represent signal of an optical slice of 1.0 µm z-thickness. Optical settings were identical for all images and master gain settings were optimized for each individual image, in which control pictures were always imaged with identical or higher master gain settings than the experimental pictures (Supplementary Table 1).

Fluorescent metabolic tracing TLC

The fluorescent metabolic tracing procedure was performed according to the click-chemistry-based method of fatty acid metabolism separation described by Thiele et al.[4] RAW264.7 macrophage cells were pre-incubated for 4h with 1.0 µg/mL LPS, after which fresh medium containing 1.0 µg/mL LPS or 1.0 µg/mL LPS and either vehicle (0.1% EtOH) or DHEA probe **3** (1 or 10 µM) were added for another 4h. Cells were grown and incubated in 100 mm aluminum foil covered culture dishes. Per condition two culture dishes containing approximately 15 million cells per dish were used. After 4h of incubation with the probe , cells were scraped in 1 mL PBS and transferred to 2 mL Eppendorf vials (330 µL per vial). Methanol (600 µL) and chloroform (150 µL) were added and the vials were briefly mixed on a vortex shaker to obtain a single liquid phase. Hereafter, the samples were centrifuged at 14000xg for 2 min, and the supernatant was transferred to a new 2 mL Eppendorf vial. Chloroform (300 µL) and 0.1% aqueous acetic acid (600 µL) were added followed by mixing on a vortex shaker and centrifugation at 14000xg for 5 min. The upper aqueous phase was discarded. The lower organic phases of each condition were combined in a new 1.5 mL Eppendorf vial and concentrated in a speedyvac concentrator at 35 °C for a maximum of 2h. The dried lipid pellets were redissolved in 7 µL chloroform, and 30 µL of click reaction mixture (5 µL of 44.5 mM 3-

azido-7-hydroxycoumarin, 500 µL of 10 mM $[ACN]_4CuBF_4$ in ACN, and 2 mL ethanol) was added. The samples were incubated and reacted in a heating block (Eppendorf Thermomixer, 24x 1.5mL block, 42 ^{o}C, without shaking) for 3h. After heating the samples were briefly centrifuged, and the lipids were redissolved by mixing for 1 min at 42 ^{o}C. The samples were applied onto a 5 x 10 cm silica TLC plate (254 nm UV-indicator, Merck No. 1.05719.0001). The plate was developed in a two-step procedure starting with $CHCl_3$:MeOH:H_2O:AcOH 65:25:4:1 (v/v/v) for 5 cm, dried for 2 min by gently heating with air, and then developed a second time for 9 cm in hexane:ethyl acetate 1:1 (v/v). The TLC plate was dried by gentle heating with air, and subsequently soaked for 5 sec in 4% ammonium hydroxide in acetone. The TLC plate was placed in a hood for 1 min to evaporate excess solvent, followed by imaging using a 366 nm UV lamp for TLC. For iodine staining, the TLC plate was placed in an iodine vaporized container.

Supplemental figures

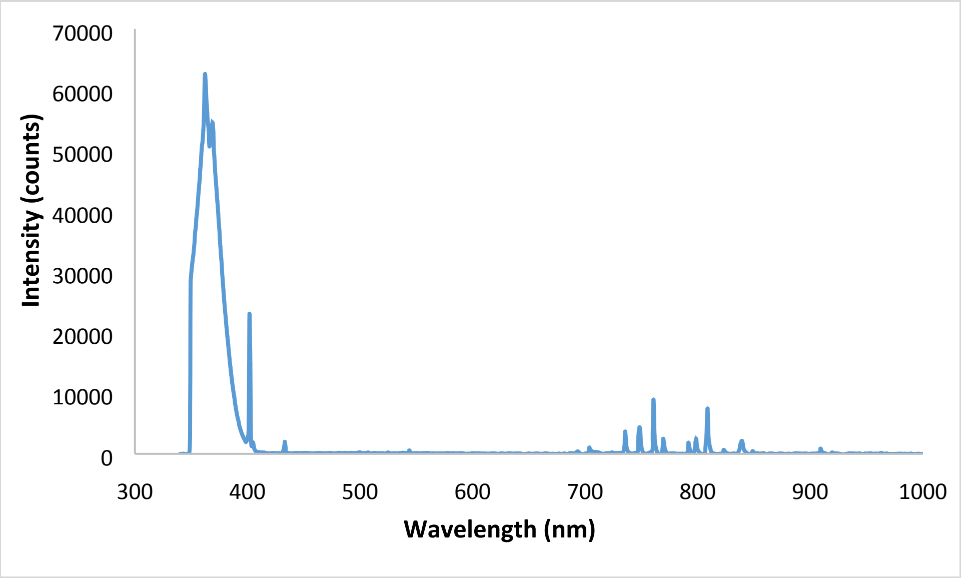

Supplementary Figure 1 Spectral output of UVP-C1000 crosslinker equipped with 5x 8W light bulbs of 366 nm. Spectral output was analyzed, using a Flame Miniature Spectrometer from Ocean Optics.

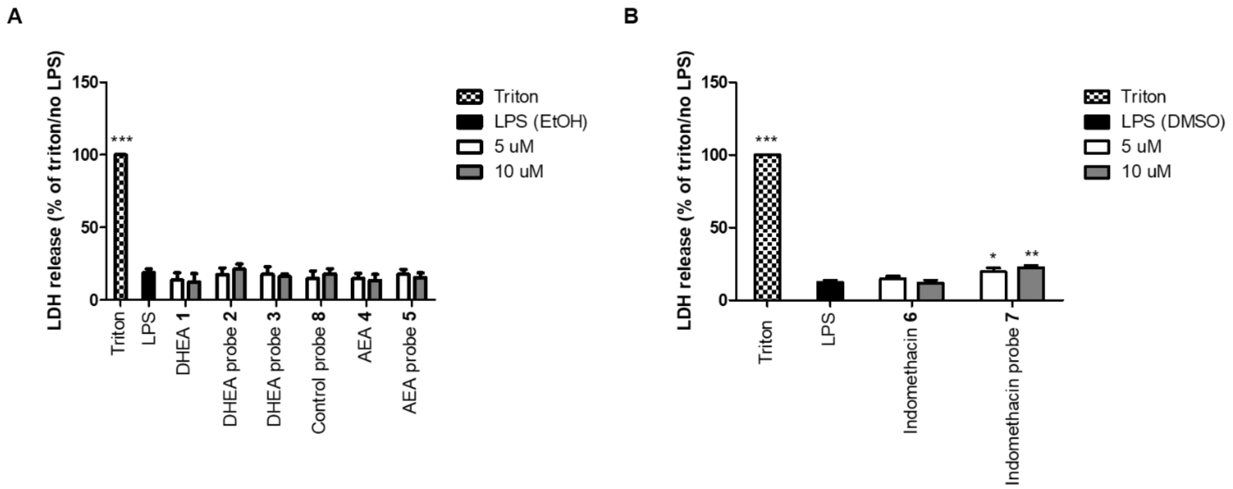

Supplementary Figure 2 LDH release of 1.0 µg mL^{-1} LPS-stimulated RAW264.7 macrophages incubated with 5.0 and 10.0 µM of PUFA-derivatives or indomethacin. A) LDH release of DHEA, AEA, and their respective synthetic bi-functional chemical probes. B) LDH release of indomethacin and its respective synthetic bi-functional chemical probe (B). The LDH release is expressed as percentage compared to 1.0% Triton X-100 treated positive control (set as 100% cytotoxicity), and non LPS-stimulated macrophages (set as 0% cytotoxicity). All samples were measured in triplicate containing technical duplicates. Asterisks indicate significant differences from the vehicle with 1.0 µg mL^{-1} LPS control (One-way ANOVA, Dunnett's multiple comparison test *post hoc*; * P<0.05, ** P<0.01, *** P<0.001).

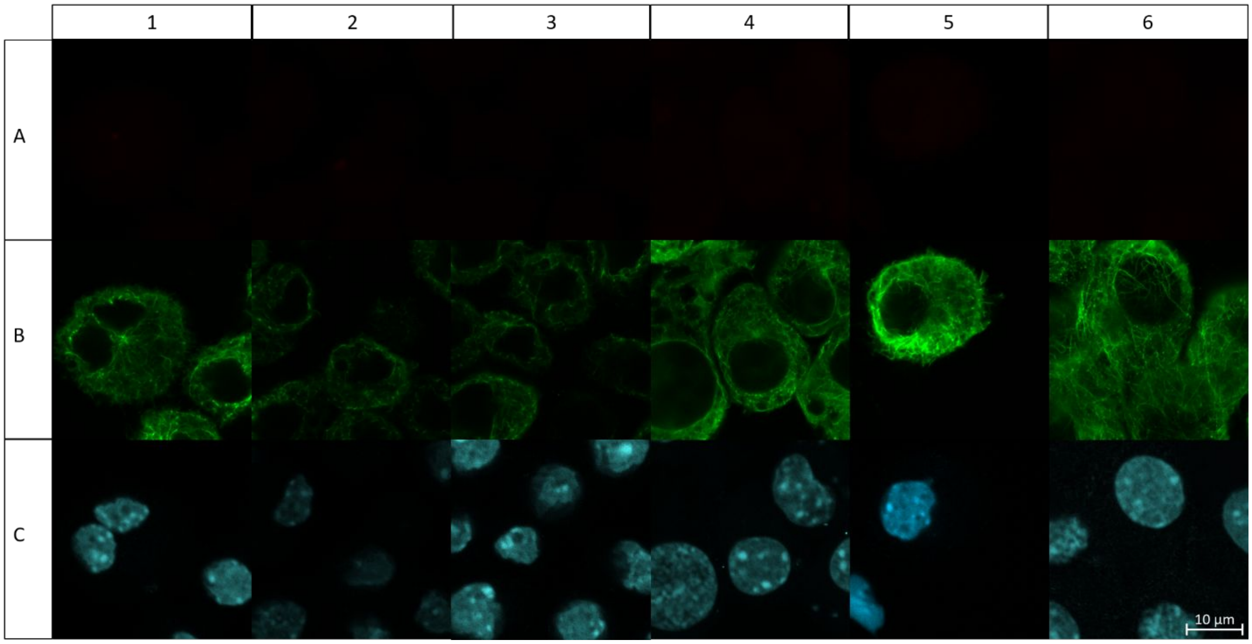

Supplementary Figure 3 Confocal microscopy images of 1.0 µg mL^{-1} LPS-stimulated RAW264.7 macrophages. A) shows the lissamine rhodamine B channel without tetrakis(acetonitrile)copper(I) hexafluorophosphate. B) shows immunostaining of alpha tubulin. C) shows DAPI staining in nuclei. 1) 10 µM DHEA probe 2. 2) 10 µM DHEA probe 3. 3) 10 µM AEA probe 5. 4) 10 µM indomethacin probe 7. 5) 10 µM probe 8. 6) 0.1% EtOH vehicle. Scale bar applies to all images in the figure.

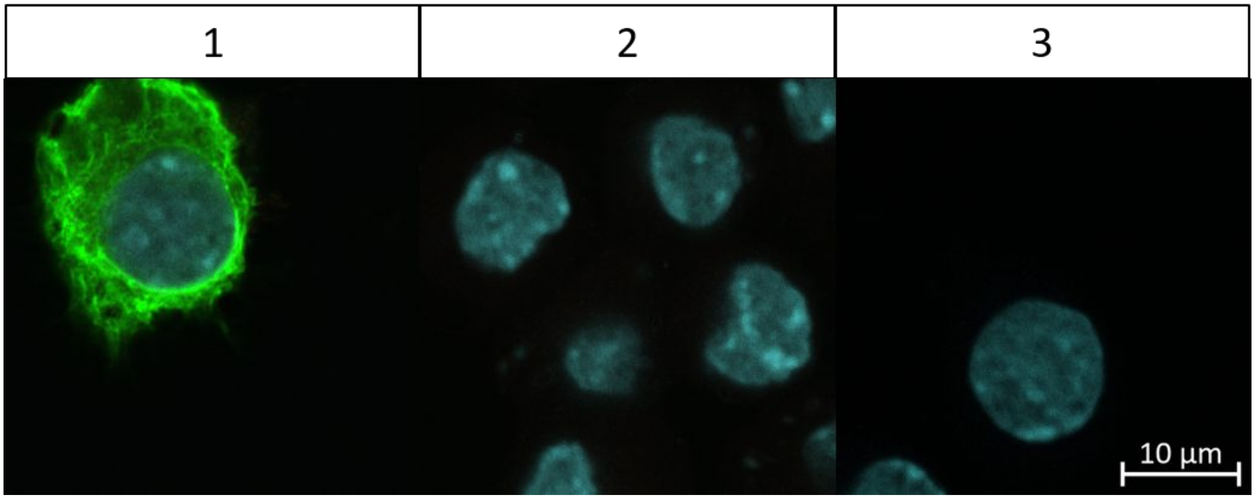

Supplementary Figure 4 Fluorescent microscopy pictures of 1.0 µg mL^{-1} LPS-stimulated RAW264.7 macrophages. Shown are overlays of the lissamine rhodamine B channel with the addition of tetrakis(acetonitrile)copper(I) hexafluorophosphate (red), Alexafluor 488 immunostaining of tubulin (green), and DAPI staining of the nuclei (blue). 1) EtOH vehicle with primary tubulin antibody. 2) 1 EtOH vehicle without primary tubulin antibody. 3) LPS stimulated cells only. Scale bar applies to all images in the figure.

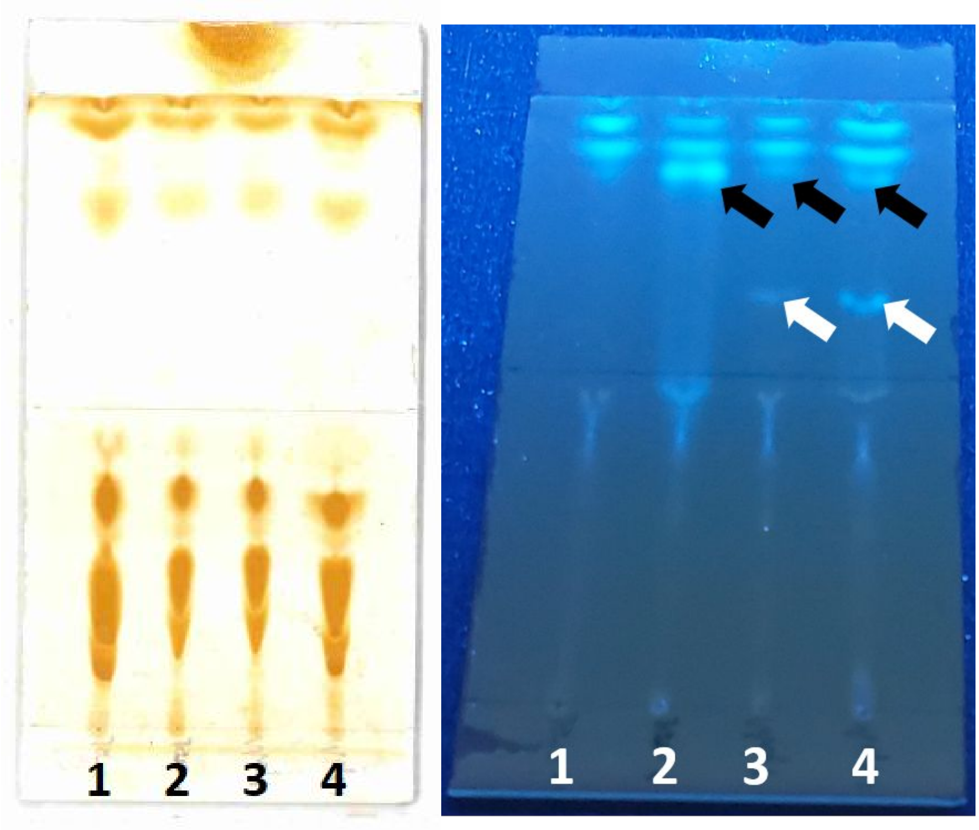

Supplementary Figure 5 Chromatogram of lipid mixture from RAW264.7 macrophage cell extracts visualized with iodine staining (left) or at 366 nm (right) (the line indicates an R_f-value of 0.5). 1) LPS treated cells (negative control), 2) LPS treated cells, spiked with 25 µM DHEA probe **3** (positive control), 3) cells treated with 1 µM DHEA probe **3**, 4) cells treated with 10 µM DHEA probe **3**. All samples were reacted with click reagent for 3h at 42 ^{0}C prior to loading on TLC. Black arrows indicate the presence of intact DHEA probe 3, white arrows indicate the single DHEA probe 3 metabolite fraction. The phospholipid components of the membrane elute with a R_f-value <0.5, the absence of fluorescence indicates that the lipid tail of DHEA probe **3** was not incorporated into components of this fraction.

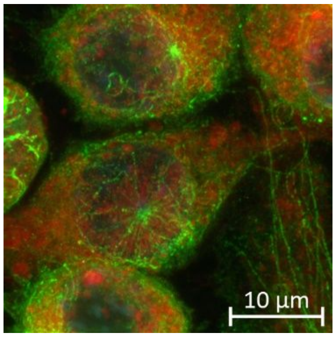

Supplementary Figure 6 Confocal fluorescent maximum intensity Z-stack projection of 1.0 µg mL^{-1} LPS-stimulated RAW264.7 macrophages incubated with 10 µM indomethacin probe **7**. Overlay shows the lissamine rhodamine B channel after the addition of tetrakis(acetonitrile)copper(I) hexafluorophosphate (red), Alexafluor 488 (AF488) immunostaining of tubulin (green), and DAPI staining of the nuclei (blue).

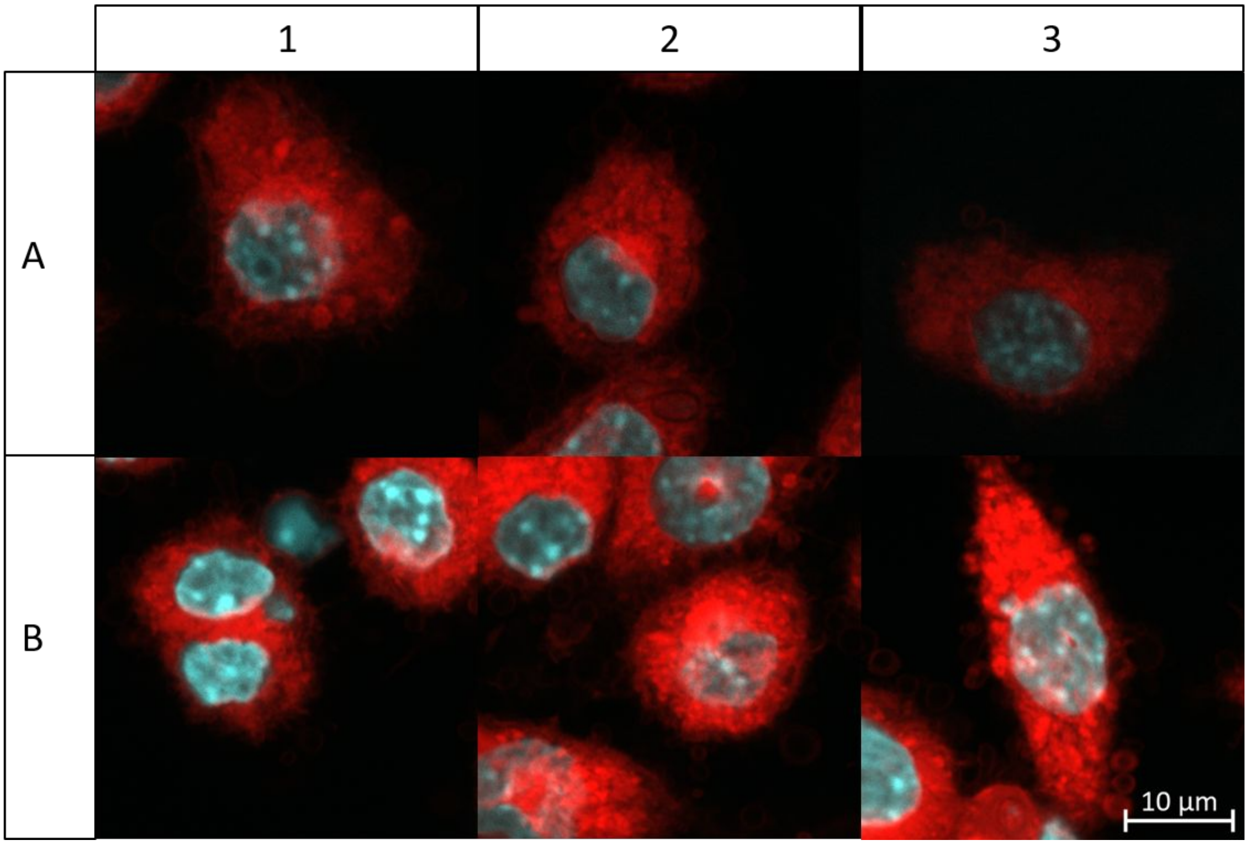

Supplementary Figure 7 Fluorescent microscopy pictures of 1.0 µg mL^{-1} LPS-stimulated RAW264.7 probe incubated macrophages. A) Shows 4 h incubation with 10 µM DHEA probe **3**, B) shows 4 h incubation with 10 µM AEA probe **5**. Overlays represent lissamine rhodamine B (red), Alexafluor488 antibody (green), and DAPI staining (blue). Cells were stained with 1) secondary AF488 antibody without primary Rac1 antibody, 2) Rab5c antibody, or 3) Ptgs2 antibody. Scale bar applies to all images in the figure.

Supplemental Tables

Supplementary Table 1 Overview of master gain settings during confocal imaging of the fluorophores.

**Figure 3**	**1**	**2**	**3**	**4**	**5**	**6**
**A** (Rhodamine)	380	450	420	450	550	550
**B** (AF488)	500	500	480	480	500	480
**C** (DAPI)	600	590	650	600	600	600

**Figure S3**	**1**	**2**	**3**	**4**	**5**	**6**
**A** (Rhodamine)	550	550	550	550	550	550
**B** (AF488)	500	460	480	480	500	480
**C** (DAPI)	600	600	650	600	640	600

**Figure S4**	**1**	**2**	**3**
Rhodamine	550	550	550
AF488	480	480	500
DAPI	600	600	600

**Figure 5**	**1**	**2**	**3**
**A** Rhodamine	500	500	500
AF488	600	600	600
DAPI	600	600	600
**B** Rhodamine	450	450	450
AF488	600	600	600
DAPI	550	550	550

**Figure S5**	
**A** Rhodamine	600
AF488	500
DAPI	507

**Figure S6**	**1**	**2**	**3**
**A** Rhodamine	500	500	500
AF488	600	600	600
DAPI	600	600	600
**B** Rhodamine	450	450	430
AF488	600	600	600
DAPI	550	550	530

Supplementary Table 2 Medium concentrations of the inflammatory markers PGE_2, IL-6, and cytotoxicity marker LDH in 1.0 µg mL^{-1} LPS-stimulated RAW264.7 macrophages incubated with 5.0 µM and 10.0 µM of DHEA, AEA, indomethacin, or their respective synthetic bi-functional chemical probe. LDH concentration is expressed as percentage compared to 1.0% Triton X-100 treated positive control (set as 100% cytotoxicity), and non LPS-stimulated macrophages (set as 0% cytotoxicity). All samples were measured in triplicate containing technical duplicates. Asterisks indicate significant differences from the 1.0 µg mL^{-1} LPS-stimulated vehicle incubation (One-way ANOVA, Dunnett's multiple comparison test *post hoc*; * P<0.05, ** P<0.01, *** P<0.001). † Value represented is an estimation of PGE_2 concentration, because absolute absorbance values were too low to be accurately determined. <LOD represent values below the calibration.

	**PGE_2 (ng mL^{-1})**	**IL-6 (ng mL^{-1})**	**LDH activity (%)**
Vehicle (0.1% EtOH)	3.62 (± 1.26)	23.77 (± 8.94)	18.9 (± 2.5)
DHEA **1** (5.0 µM)	[redacted]	16.71 (± 8.56)	13.8 (± 5.0)
DHEA **1** (10.0 µM)	0.40 (± 0.10) ***	14.00 (± 5.30)	12.4 (± 5.8)
DHEA probe **2** (5.0 µM)	[redacted]	10.64 (± 4.27)	17.6 (± 4.5)
DHEA probe **2** (10.0 µM)	0.16 (± 0.03) ***	10.53 (± 3.85)	21.2 (± 3.6)
DHEA probe **3** (5.0 µM)	[redacted]	13.47 (± 7.87)	17.7 (± 5.2)
DHEA probe **3** (10.0 µM)	0.27 (± 0.05) ***	7.66 (± 4.31) *	16.2 (± 1.8)
AEA **4** (5.0 µM)	[redacted]	27.11 (± 10.26)	14.9 (± 3.4)
AEA **4** (10.0 µM)	[redacted]	29.19 (± 10.90)	13.6 (± 4.1)
AEA probe **5** (5.0 µM)	[redacted]	15.01 (± 14.32)	17.8 (± 3.3)
AEA probe **5** (10.0 µM)	[redacted]	12.18 (± 9.65)	15.5 (± 3.2)
Control probe **8** (5.0 µM)	[redacted]	21.51 (± 6.83)	14.9 (± 5.2)
Control probe **8** (10.0 µM)	*2.6 (± 0.8)†*	21.84 (± 9.43)	18.1 (± 3.5)
Vehicle (0.1% EtOH) without LPS	< LOD ***	< LOD ***	0
	**PGE_2 (pg mL^{-1})**	**IL-6 (ng mL^{-1})**	**LDH activity (%)**
Vehicle (0.1% DMSO)	2.95 (± 0.72)	18.65 (± 6.84)	12.3 (± 4.3)
Indomethacin **6** (5.0 µM)	[redacted]	16.49 (± 8.73)	14.8 (± 4.4)
Indomethacin **6** (10.0 µM)	< LOD ***	14.96 (± 6.94)	11.9 (± 5.3)
Indomethacin probe **7** (5.0 µM)	[redacted]	14.23 (± 5.47)	19.7 (± 6.4) *
Indomethacin probe **7** (10.0 µM)	< LOD ***	15.15 (± 6.66)	22.6 (± 3.7) **
Vehicle (0.1% DMSO) without LPS	< LOD ***	< LOD ***	0

Synthesis of chemical probes

Materials

Ammonia (ca. 7N solution in MeOH), 2-(3-(but-3-yn-1-yl)-3H-diazirin-3-yl)ethan-1-amine (≥95%), celite® 545, chloroform-d (≥99.8 atom % D, contains 0.5 wt % silver foil as stabilizer, 0.03% (v/v) TMS), distilled triethylamine (TEA) (>99.5%), hydroxylamine-O-sulfonic acid (≥97%), iodine (≥99%), propargyl-N-hydroxysuccinimidyl ester, ammonium bicarbonate (≥99%), 1,2,4,5-tetrachloro-3-nitrobenzene (Standard for quantitative NMR, TraceCERT®) were purchased from Sigma-Aldrich (Zwijndrecht, The Netherlands). 4-Amino-2-butanol (≥98%), bis(2-oxo-3-oxazolidinyl)phosphinic chloride (BOP-Cl) (≥97%), di-tert-butyl dicarbonate (≥97%), isobutyl chloroformate (≥98%), methanol Extra Dry over Molecular Sieves (≥99.8%), oxalyl chloride (≥98%), sodium sulphate (≥99%), sodium thiosulphate (≥98.5%) were purchased from Fisher Scientific (Landsmeer, The Netherlands). Dimethyl sulfoxide (DMSO) (≥98%) was purchased from TCI Chemicals (Zwijndrecht, Belgium). Acetonitrile (≥ 99.9%, HiPerSolv CHROMANORM® for LC-MS), ethyl acetate (EtOAc) (technical grade) heptane (technical grade), silica gel 40-63 µm for flash chromatography, sodium dodecyl sulfate (SDS) (≥ 99%, Biotechology grade) were purchased from VWR Chemicals (Amsterdam, The Netherlands). Formic acid (≥99%, ULC/MS grade, and trifluoroacetic acid (≥99.95%, HPLC grade) were obtained from Biosolve B.V. (Valkenswaard, The Netherlands). Ethanol (absolute for analysis EMSURE®), was obtained from Merck (Amsterdam, The Netherlands). Pure chloroform-*d* (100.0 atom% D) was obtained from Janssen Chimica (Beerse, Belgium). Anhydrous DCM and THF were obtained using a Pure Solv 400 solvent purification system from Innovative Technology (Amesburry, USA). Ultrapure water was filtered by a MilliQ integral 3 system from Millipore (Molsheim, France).

Synthesis of *tert*-butyl 3-oxobutylcarbamate (c)

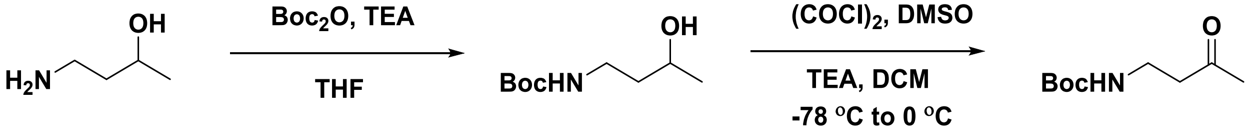

a b c

An oven-dried round bottom flask (RBF) containing 40 mL anhydrous tetrahydrofuran (THF) was placed under argon, and 1.7 mL (13 mmol, 1.2 eq.) freshly distilled triethylamine (TEA) and 1.0 mL (10 mmol, 1.0 eq.) 4-amino-2-butanol **a** were added. While stirring the solution continuously at room temperature, 2.5 g (12 mmol, 1.1 eq.) of Boc_2O was added in small portions over a time span of 10 min. After stirring the reaction mixture for 1 h at room temperature the reaction was quenched by pouring the mixture in 50 mL ice cold 0.5 M HCl. Extraction of the product with 3 x 100 mL EtOAc was performed, the organic layers were combined and dried with anhydrous Na_2SO_4 to obtain *tert*-butyl (3-hydroxybutyl)carbamate **b**.

An oven-dried three-necked RBF with 200 mL of anhydrous dichloromethane (DCM) was placed under argon atmosphere and cooled to -78 °C. Then, 1.8 mL (21 mmol, 2 equiv.) oxalyl chloride was added, followed by drop-wise addition of 3.0 mL (42 mmol, 4 eq.) dimethyl sulfoxide (DMSO). The reaction was stirred for 30 min at -78 °C. *Tert*-butyl (3-hydroxybutyl)carbamate **b** was dissolved in 20 mL anhydrous DCM and subsequently added to the reaction over a period of 25-30 min. After stirring the reaction for 2 h at -78 °C, 5.8 mL (42 mmol, 4 eq.) of freshly distilled TEA was added to the reaction which was stirred for another 2 h at -78 °C. Then, the temperature was brought to 0 °C by placing the RBF in an ice-bath for 30 min, and was then warmed to room temperature. Hereafter, the reaction mixture was diluted with 220

mL Et_2O and passed through a silica plug. The remaining mixture was concentrated under reduced pressure, providing a yellow oil. The final product was obtained by purification of the residue by SiO_2 flash chromatography, using 50% EtOAc/heptane, providing the title compound **c** as a slightly yellowish oil (1.6 g, 81%); ^1H NMR (400 MHz, $CDCl_3$) δ 4.99 (s, 1H), 3.34 (q, *J* = 6.0 Hz, 2H), 2.66 (t, *J* = 5.8 Hz, 2H), 2.15 (d, *J* = 1.0 Hz, 3H), 1.42 (s, 9H). ^{13}C NMR (101 MHz, $CDCl_3$) δ 208.5, 156.1, 79.5, 43.8, 35.4, 30.4, 28.7.; MS (ESI+) *m/z* calculated for $C_9H_{17}NO_3$ $[M+H]^+$ 188.12812, found 188.12766, and for $[M+Na]^+$ 210.11006, found 210.10978.

Synthesis of *tert*-butyl (2-(3-methyl-3H-diazirin-3-yl)ethyl)carbamate (e)

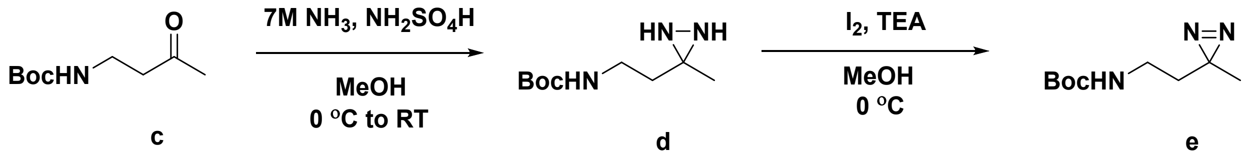

In a three-necked RBF 1.56 g (8.33 mmol, 1 eq.) *tert*-butyl(3-oxobutyl)carbamate **c** was dissolved in 18.8 mL 7.0 N NH_3 in methanol at 0 °C under argon. This mixture was stirred at 0 °C for 3 h, after which a solution of hydroxylamine-O-sulfonic acid 1.09 g (9.58 mmol, 1.15 eq.) in 18.8 mL anhydrous MeOH was added drop-wise in 20 min. After allowing the reaction to slowly warm to room temperature overnight, the reaction mixture was concentrated under a stream of nitrogen. The residue was re-dissolved in 18.8 mL Et_2O and passed through a celite pad. The filtrate was concentrated under reduced pressure and re-dissolved in 9.4 mL anhydrous MeOH in an amberized RBF. The RBF was placed under argon and the mixture was cooled to 0 °C. While stirring, 1.7 mL (13 mmol, 1.5 eq.) freshly distilled TEA was added after which the reaction was allowed to stir for 5 min. Following, iodine 2.35 g (9.16 mmol, 1.1 eq.) was slowly added in small portions over a period of 30 min until a red-brown color persisted, which was checked by taking a small droplet from the mixture with a pipette. After iodine addition the reaction mixture was stirred for 30 min at 0 °C the reaction was quenched with 94 mL saturated $Na_2S_2O_3$ solution. The quenched reaction mixture was vigorously mixed for 10 min and 188 mL EtOAc was added. The product was extracted with 3 x 100 mL EtOAc and the combined organic layers were dried using anhydrous Na_2SO_4. The remaining mixture was concentrated under reduced pressure. The title product **e** was purified by SiO_2 flash chromatography using 35% EtOAc/heptane, providing the final product as a yellow oil (0.45 g, 27%); ^1H NMR (400 MHz, $CDCl_3$) δ 4.56 (s, 1H), 3.05 (q, *J* = 6.7 Hz, 2H), 1.56 (t, *J* = 6.9 Hz, 2H), 1.45 (s, 9H), 1.05 (s, 3H); ^{13}C NMR (101 MHz, $CDCl_3$) δ 79.7, 35.9, 34.8, 29.2, 28.5, 20.0. MS (ESI+) *m/z* calculated for $[M+H]^+$ $C_9H_{17}O_2N_3$ 200.13902, found 200.13898, and for $[M+Na]^+$ 222.12130, found 222.12087.

Synthesis of diazirine amine (f)

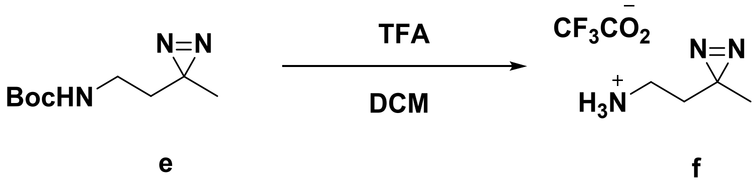

Note: The deprotection of amine was performed immediately before coupling to the docosahexaenoic acid alkyne.

In an amberized glass vial 80 mg (0.38 mmol, 1 eq.) *tert*-butyl(2-(3-methyl-3H-diazirin-3-yl)ethyl)carbamate **e** was dissolved in 0.8 mL anhydrous DCM. The solution was cooled to 0 °C while stirring, and subsequently 0.2 mL (2.6 mmol, 6.4 eq.) TFA was added to the mixture. After 1 h the reaction mixture was concentrated under a stream of N_2. The residue was dissolved in 5 mL anhydrous DCM and concentrated again for three times after which the residue was put under high vacuum for 1 h. The remaining residue was used in subsequent coupling without further purification. TLC and ^{1}H-NMR analysis were used to confirm deprotection.

Synthesis of DHEA probe 3

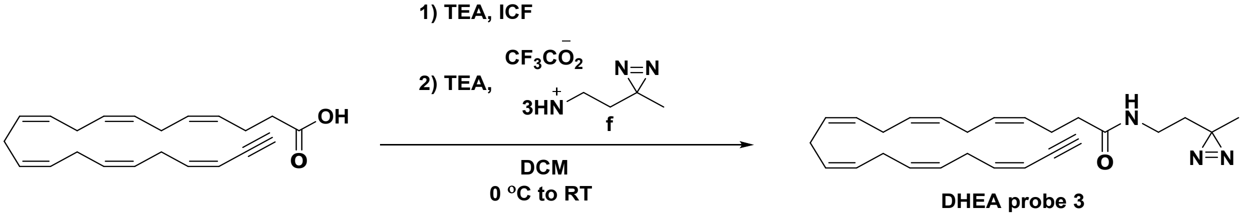

In a 3 mL glass vial, 1.0 mg (3.1 µmol, 1.0 eq.) DHA-alkyne in EtOH was evaporated to dryness using co-evaporation with DCM. After complete evaporation, the glass vial was placed under argon atmosphere in 1 mL anhydrous DCM and protected from light by aluminum foil. Then 6.0 µL of freshly distilled TEA (43 µmol, 14 eq.) was added, followed by 4.8 µL isobutyl chloroformate (37 µmol, 12 eq.) in anhydrous DCM. The reaction was allowed to stir for 1 h at room temperature after which the reaction mixture was cooled in an ice bath. Hereafter, 3.4 mg of 2-(3-methyl-3H-diazirin-3-yl)ethan-1-amine **f** (34 µmol, 11 eq.) was dissolved in 1 mL of dry DCM in an amberized glass vial together with 4.7 µL freshly distilled TEA (34 µmol, 11 eq.), and added to the reaction mixture. The reaction was stirred on ice for overnight before the solvent was evaporated under a stream of nitrogen. The reaction mix was re-dissolved in 1 mL 2/8 (water/ACN) and purified by semi-preparative HPLC (*vide infra*). Quantification was performed using quantitative NMR analysis. The title compound was synthesized as 245 µg (20% yield) pure product. ^{1}H NMR (600 MHz, $CDCl_3$) δ 5.98 (dt, *J* = 10.8, 7.4 Hz, 1H), 5.62 – 5.31 (m, 12H), 3.19 (q, *J* = 6.6 Hz, 3H, theor. 2H), 3.17 – 3.12 (m, 3H), 2.89 (dt, *J* = 21.5, 6.1 Hz, 8H), 2.44 (p, *J* = 6.9 Hz, 2H), 2.27 (t, *J* = 7.5 Hz, 2H), 1.62 (t, *J* = 6.8 Hz, 3H, theor. 2H), 1.07 (s, 3H). MS (ESI+) *m/z* calculated for $C_{26}H_{35}N_3O$ $[M+H]^+$ 406.28529, found 406.28473 and for $[M+Na]^+$ 428.26723, found 428.26592.

Synthesis of DHEA probe 2

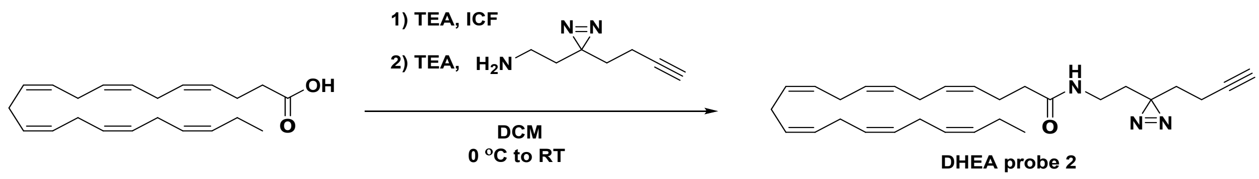

20 mg (61 µmol, 1.0 eq.) docosahexaenoic acid in EtOH was pipetted into a 5 mL glass vial, evaporated to dryness using co-evaporation with DCM, and placed under argon atmosphere. The docosahexaenoic acid was dissolved in 2 mL of anhydrous DCM, and treated with 11.8 µL freshly distilled TEA (84.7 µmol, 1.4 eq.) and 9.7 µL of isobutyl chloroformate (75 µmol, 1.2 eq.) for 1 h at room temperature. After 1 h the reaction was cooled in an ice bath and protected from light by aluminum foil. To the mixture 0.5 mL of anhydrous DCM containing 9.4 µL freshly distilled TEA (67 µmol, 1.1 eq.) and 9.2 mg of 2-(3-(But-3-yn-1-yl)-3H-diazirin-3-yl)ethan-1-amine (67 µmol, 1.1 eq.) was added. The reaction was stirred on ice for overnight. Next day the reaction mixtures were evaporated to dryness under a stream of nitrogen and dissolved in 2.0 mL 2/8 (water/ACN). The title product was purified by preparative-HPLC *vide infra*, and finally obtained as 16.0 mg (59% yield) of pure oil. ^{1}H NMR (400 MHz, $CDCl_3$) δ 5.55 (s, 1H), 5.48 – 5.23 (m, 12H), 3.10 (q, *J* = 6.5 Hz, 2H), 2.84 (q, *J* = 8.0, 6.7 Hz, 10H), 2.42 (q, *J* = 7.1 Hz, 2H), 2.23 (t, *J* = 7.5 Hz, 2H), 2.17 – 1.93 (m, 5H), 1.67 (dt, *J* = 17.9, 7.0 Hz, 4H), 0.97 (t, *J* = 7.6 Hz, 3H). ^{13}C NMR (101 MHz, $CDCl_3$) δ 172.5, 134.1–125.6 (m), 82.8, 77.4, 69.5, 36.6, 34.4, 32.7, 32.3, 27.0, 25.8, 23.5, 20.7, 14.4, 13.4.; MS (ESI+) *m/z* calculated for $C_{29}H_{41}N_3O$ $[M+H]^+$ 448.33224, found 448.33154 and for $[M+Na]^+$ 470.31418, found 470.31285.

Synthesis of AEA probe 5

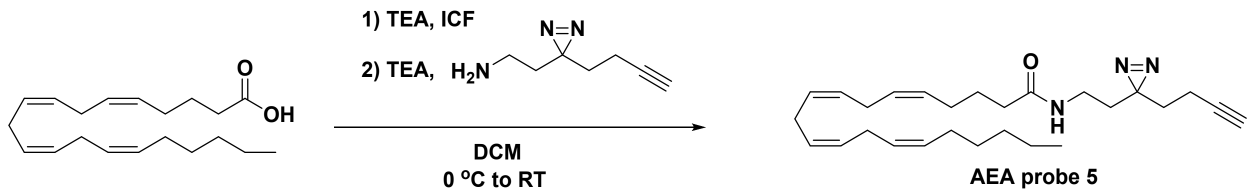

20 mg arachidonic acid in EtOH (66 µmol, 1.0 eq.) was pipetted into a 5 mL glass vial, evaporated to dryness using co-evaporation with DCM, and placed under argon atmosphere. The arachidonic acid was dissolved in 2 mL of anhydrous DCM, and reacted with 12.8 µL freshly distilled TEA (91.8 µmol, 1.4 eq.) and 10.2 µL of isobutyl chloroformate (78.6 µmol, 1.2 eq.) for 1 h at room temperature. After 1 h the reaction was cooled in an ice bath and protected from light by aluminum foil. To the mixture 0.5 mL of anhydrous DCM containing 10.1 µL freshly distilled TEA (72.5 µmol, 1.1 eq.) and 9.9 mg of 2-(3-(But-3-yn-1-yl)-3H-diazirin-3-yl)ethan-1-amine (73 µmol, 1.1 eq.) was added. After overnight stirring on ice, the reaction mixture was evaporated to dryness under a stream of nitrogen and dissolved in 2.0 mL 2/8 (water/ACN). The title product was purified by preparative-HPLC *vide infra*, and finally obtained as 13.2 mg (47% yield) of pure oil. ^{1}H NMR (400 MHz, $CDCl_3$) δ 5.51 (d, *J* = 6.4 Hz, 1H), 5.46 – 5.27 (m, 7H, theor. 8H), 3.11 (q, *J* = 6.4 Hz, 2H), 2.83 (dt, *J* = 11.3, 5.6 Hz, 6H), 2.24 – 1.98 (m, 9H), 1.78 – 1.59 (m, 6H), 1.42 – 1.23 (m, 6H), 0.89 (t, *J* = 6.8 Hz, 3H). ^{13}C NMR (101 MHz, $CDCl_3$) δ 173.3, 132.0–126.2 (m), 83.1, 69.9, 36.5, 34.7, 33.0, 32.6, 29.8, 27.7, 27.3, 27.1, 26.1, 25.9, 23.0, 14.5, 13.7. MS (ESI+) *m/z* calculated for $C_{27}H_{41}N_3O$ $[M+H]^+$ 424.33224, found 424.33248 and for $[M+Na]^+$ 446.31418, found 446.31461.

Synthesis of Indomethacin probe 7

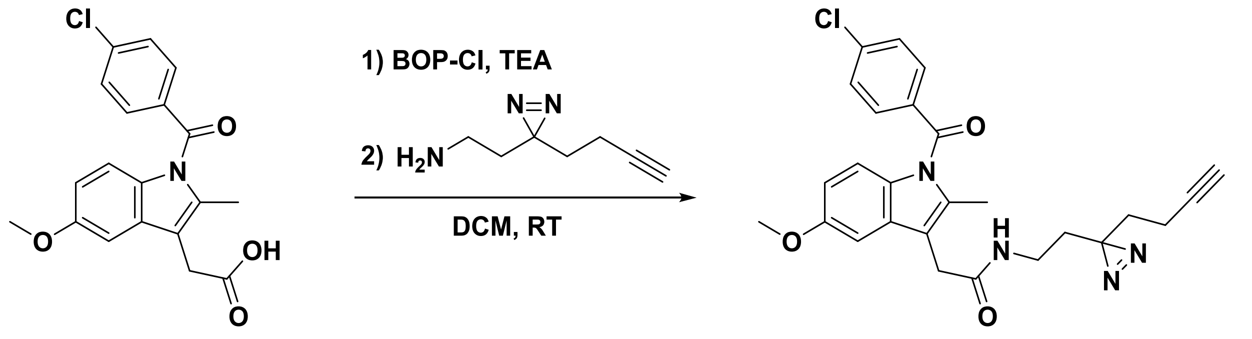

Indomethacin probe 7

For the synthesis of an alkyne and diazirine based indomethacin probe 100 mg of indomethacin (0.28 mmol, 2.6 eq.) and 71 mg (0.28 mmol, 2.6 eq.) BOP-Cl were dissolved in 5 mL anhydrous DCM in an argon filled dry amberized RBF. To the reaction 78 µL (0.56 mmol, 5.2 eq.) of freshly distilled triethylamine was added, and the reaction was stirred at room temperature. After 10 min, 15 mg 2-(3-(But-3-yn-1-yl)-3H-diazirin-3-yl)ethan-1-amine (0.11 mmol, 1 eq.) was added and stirred for over the weekend. The crude mixture was concentrated by reduced pressure and purified by SiO_2 flash chromatography using 50% EtOAc in hexanes, providing the final product as a yellow solid (14.9 mg, 29%). ^{1}H NMR (400 MHz, $CDCl_3$) δ 7.82 – 7.57 (m, 2H), 7.56 – 7.39 (m, 2H), 6.96 – 6.80 (m, 2H), 6.70 (dd, *J* = 9.0, 2.3 Hz, 1H), 5.73 (d, *J* = 6.2 Hz, 1H), 3.83 (s, 3H), 3.65 (s, 2H), 3.10 (q, *J* = 6.2 Hz, 2H), 2.42 (s, 3H), 1.95 – 1.77 (m, 3H), 1.57 (dt, *J* = 33.9, 6.8 Hz, 4H). ^{13}C NMR (101 MHz, $CDCl_3$) δ 168.3, 156.3, 139.6, 136.6, 133.6, 131.2, 131.0, 130.3, 129.2, 115.2, 112.5, 112.3, 100.9, 82.6, 69.3, 55.8, 34.7, 32.5, 32.3, 31.9, 26.8, 13.3, 13.0. MS (ESI+) *m/z* calculated for $C_{26}H_{25}ClN_4O_3$ $[M+H]^+$ 477.16879, found 477.16917 and for $[M+Na]^+$ 499.15074, found 499.15114.

Synthesis of control probe 8

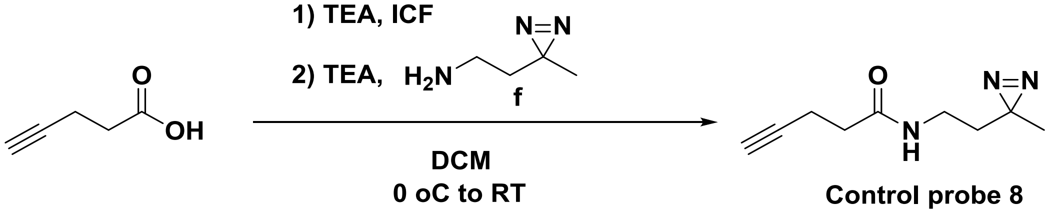

Control probe 8

20 mg 4-pentynoic acid (0.20 mmol, 1.0 eq.) was weighted and placed under argon in an amberized 3-necked RBF. The 4-pentynoic acid was dissolved in 10 mL anhydrous DCM, and subsequently 40 µL freshly distilled triethylamine (0.29 mmol, 1.4 eq.) and 32 µL isobutyl chloroformate (0.25 mmol, 1.2 eq.) were added. The reaction was stirred at room temperature for 1 h and then cooled on ice. To the cooled reaction mixture, 31 µL (0.22 mmol, 1.1 eq.) of freshly distilled triethylamine and 22 mg (0.22 mmol, 1.1 eq.) of 2-(3-methyl-3H-diazirin-3-yl)ethan-1-amine **f** were added. The reaction was stirred on ice for overnight. Next day 20 mL water was added and the resulting mixture was stirred for 10 min, after which the phases were separated. The water layer was extracted with extra 3x 25 mL EtOAc, and the combined organic layers were finally extracted with brine and dried using Na_2SO4. The mixture was evaporated to dryness and dissolved in 3 mL 2:1 (ACN:water). Purification was performed using preparative HPLC (*vide infra*) and the title compound was finally obtained as 6.9 mg (19% yield) of pure oil. ^{1}H NMR (400 MHz,

$CDCl_3$) δ 5.65 (s, 1H), 3.20 (qd, *J* = 6.8, 1.8 Hz, 2H), 2.54 (td, *J* = 7.1, 2.6 Hz, 2H), 2.46 – 2.29 (m, 2H), 2.01 (q, *J* = 2.4 Hz, 1H), 1.62 (td, *J* = 6.8, 1.7 Hz, 2H), 1.06 (d, *J* = 1.7 Hz, 3H). ^{13}C NMR (101 MHz, $CDCl_3$) δ 171.0, 82.9, 69.5, 35.4, 34.7, 34.1, 24.4, 19.8, 14.8. MS (ESI+) *m/z* calculated for $C_9H_{13}N_3O$ $[M+H]^+$ 180.11314, found 180.11284, and for $C_9H_{13}N_3O$ $[M+Na]^+$ 202.09508, found 202.09460.

Purification and quantification of chemical probes

Preparative HPLC purification of DHEA probe 2 and AEA probe 5

The desired DHEA probe **2** & AEA probe **5** were purified by preparative-HPLC on an Agilent 1260 Preparative HPLC with DAD and MSD using a PrepHT XDB-C18 21.2x250 mm, 7µ column (Agilent Technologies, Amstelveen, The Netherlands). Purification was performed using a flow rate of 20 mL min^{-1} with solvent A being water containing 0.1% FA and solvent B being ACN containing 0.1% FA. The gradient profile started with 5 min of 80% B in A, followed by a linear increase to 100% B at min 13. The run was continued isocratically at 100% B until min 18, after which the gradient was switched back to 80% B in A in 0.5 min. The run was continued at 80% B in A until min 23. The collected fractions were evaporated using speedyvac concentrator (Salm and Kipp, Breukelen, The Netherlands).

Semi-Preparative HPLC purification of DHEA probe 3

The desired DHEA probe **3** was purified by semi-preparative-HPLC on an Agilent 1260 Preparative HPLC with DAD and MSD using a Semi-Prep Zorbax Eclipse XDB-C18 9.4 x 250 mm, 5µ column (Agilent Technologies, Amstelveen, The Netherlands). The purification was performed using an isocratic run of 20% water in ACN with 0.1% FA at a flowrate of 4.0 mL min^{-1}. After purification the title molecule was concentrated under a stream of nitrogen followed by lyophilization.

Preparative HPLC purification of control probe 8

The desired control probe **8** was purified by preparative-HPLC on an Agilent 1260 Preparative HPLC with DAD and MSD (Agilent Technologies, Amstelveen, The Netherlands) using a Grace Alltima C18 5µ 250 mm x 22 mm column (Fisher Scientific, Landsmeer, The Netherlands). The sample was dissolved in 3 mL (2:1 ACN:MQ +0.1%FA), and filtered through a syringe filter to remove insoluble precipitates. Then the product was purified by HPLC purification using gradient elution. Gradient elution was performed using a flow rate of 16 mL min^{-1} with solvent A being water containing 0.1% FA and solvent B being ACN containing 0.1% FA. The gradient started with 5 min of 5% B in A, followed by a linear increase to 95% B in A which was achieved at min 20. The gradient was kept at 95% B in A until min 23, before the gradient was returned to 5% B in A in 0.5 min. The column was re-equilibrated for until min 26 at 5% B in A. After purification the title molecule was concentrated under a stream of nitrogen followed by lyophilization.

Quantification of DHEA probe 3

Quantification of DHEA probe **3** was performed using quantitative NMR. Deuterated chloroform containing 0.03% TMS was used as internal standard being placed in Wilmad® coaxial insert (Sigma Aldrich, Zwijdrecht, The Netherlands). Calibration of the signal was performed using 1,2,4,5-Tetrachloro-3-nitrobenzene (Standard for quantitative NMR, TraceCERT®) in concentrations ranging from 3.8-0.9 mM. DHEA probe **3** was quantified based on the calibration of 1,2,4,5-Tetrachloro-3-nitrobenzene (see figure S21-23).

NMR spectra

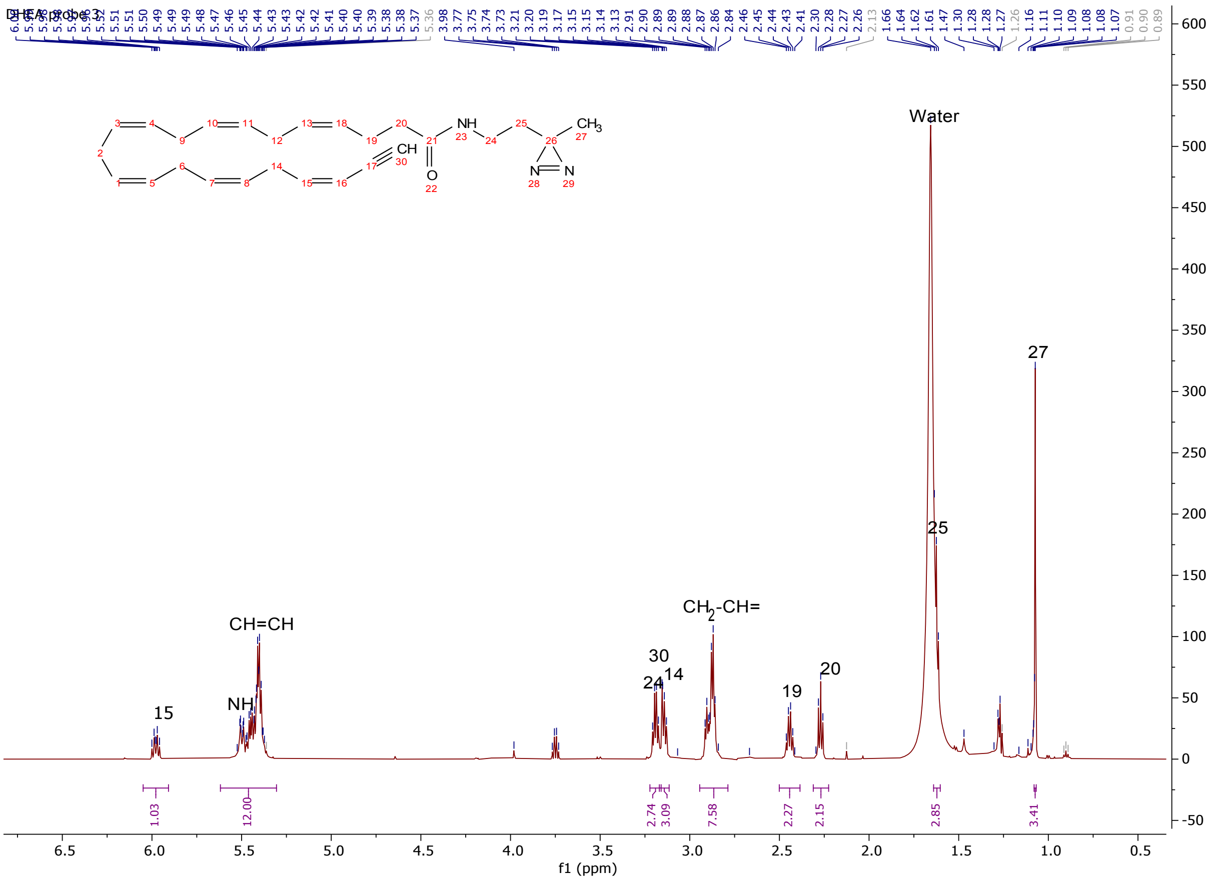

Supplementary Figure 8 ^{1}H NMR spectrum DHEA probe **3**

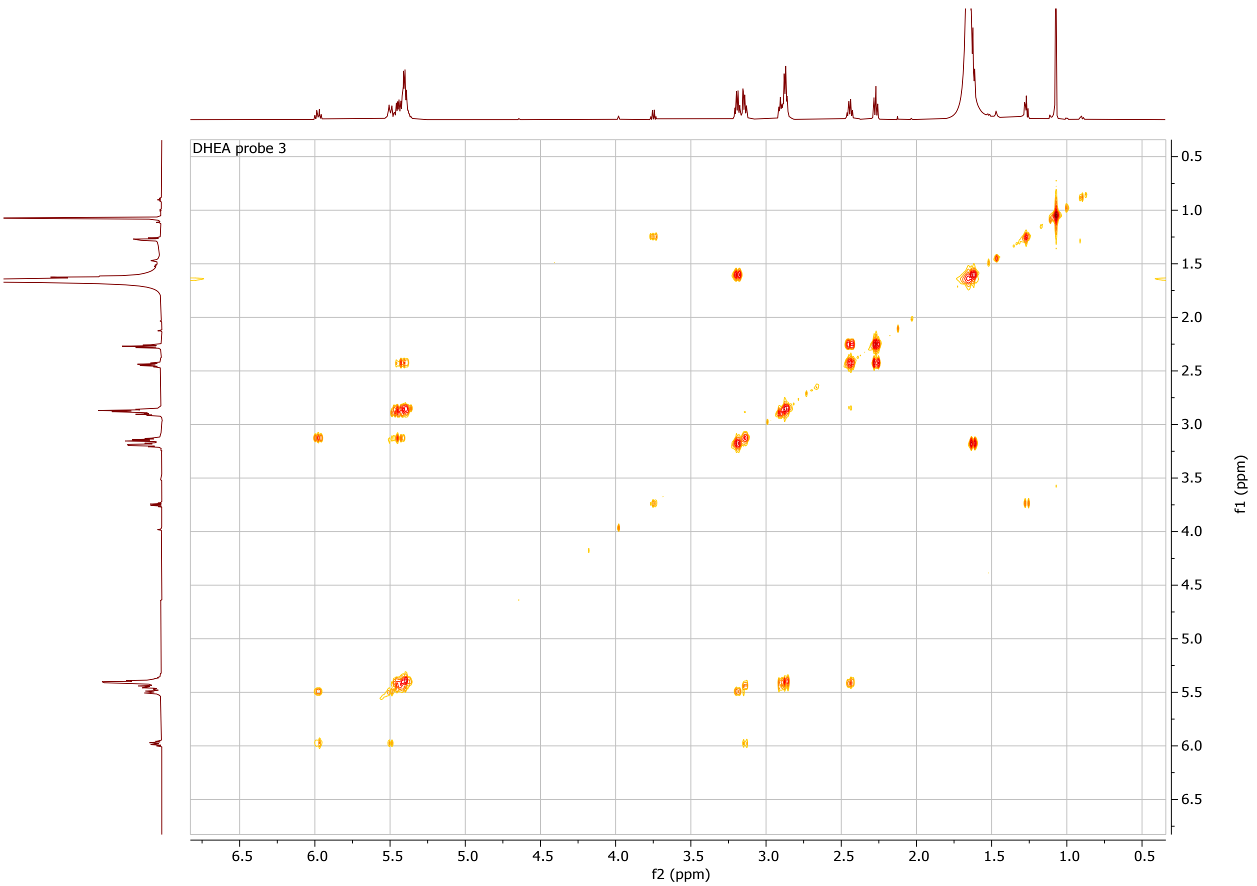

Supplementary Figure 9 COSY spectrum of DHEA probe **3**

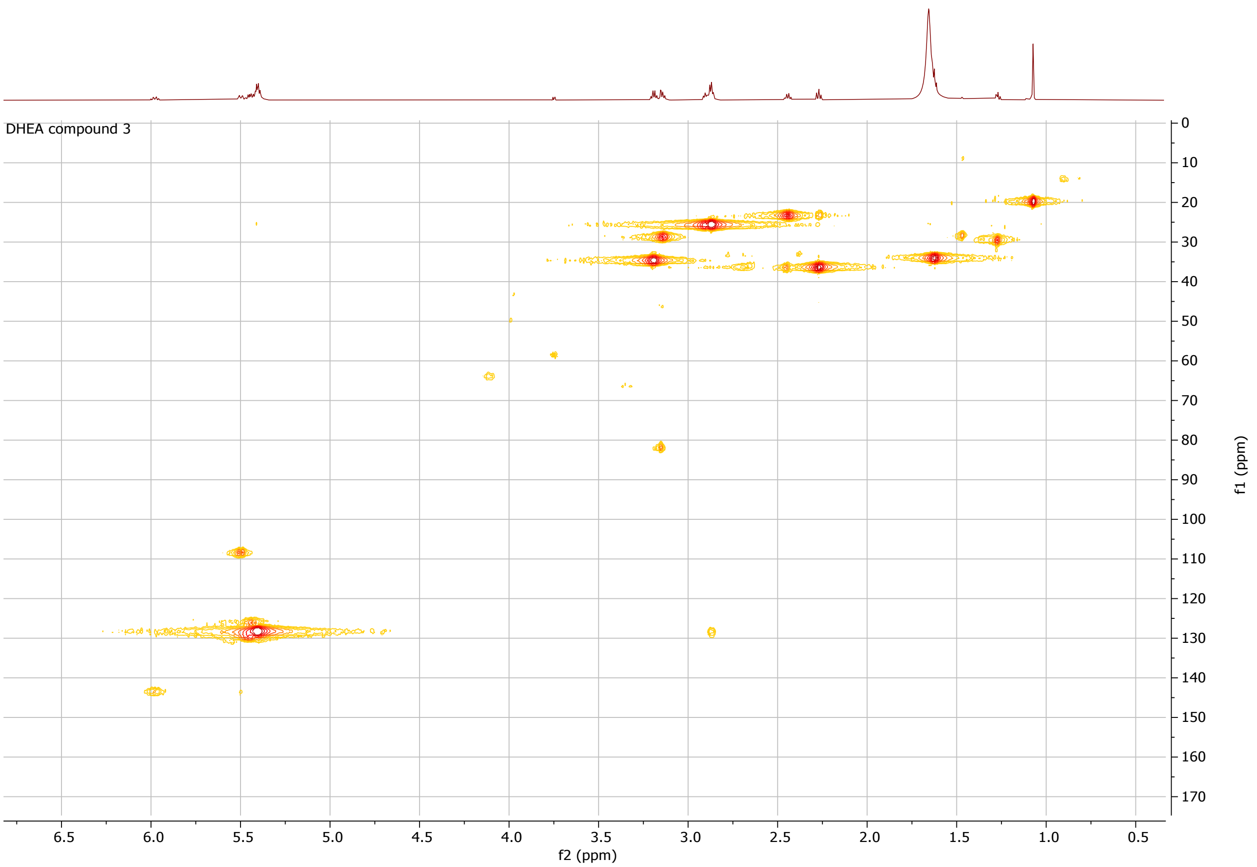

Supplementary Figure 10 HSQC spectrum of DHEA probe **3**

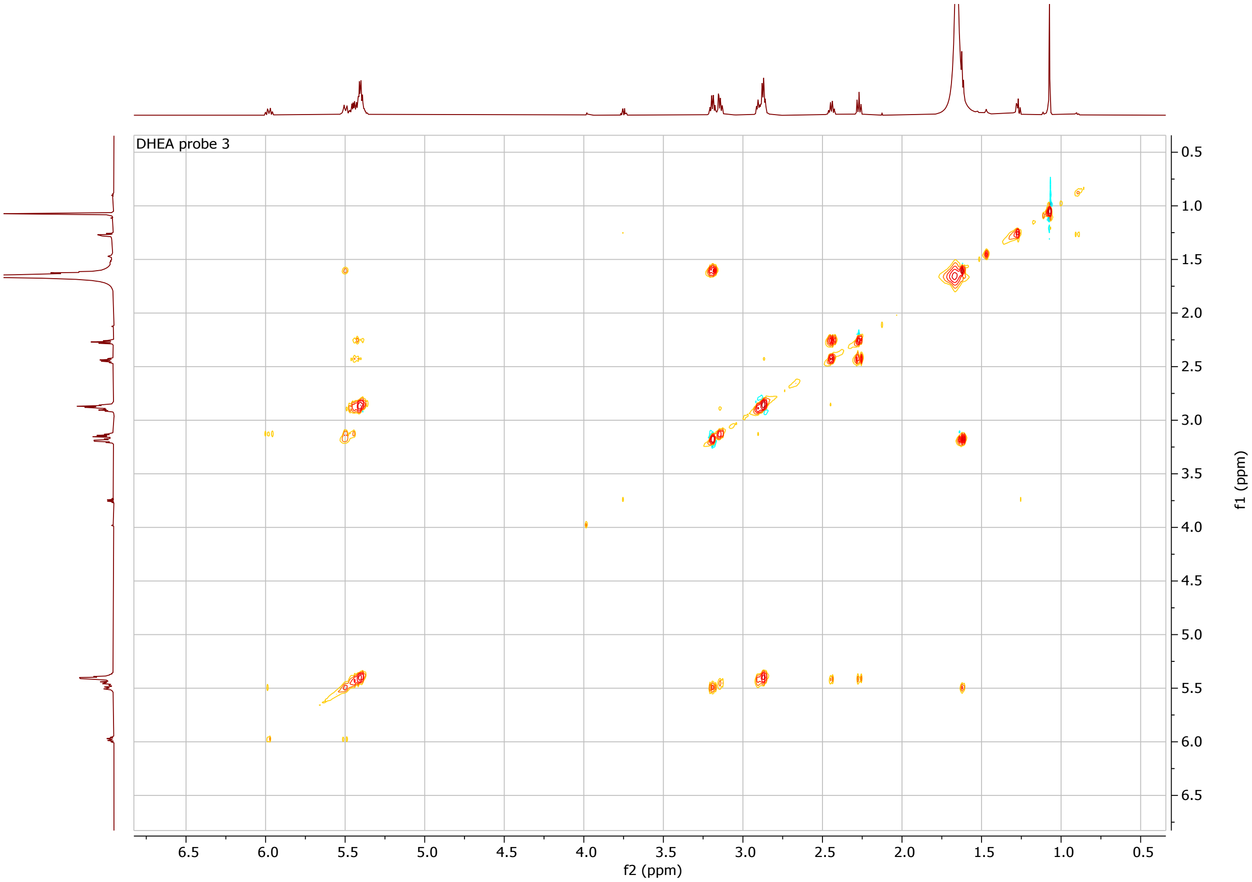

Supplementary Figure 11 TOCSY spectrum of DHEA probe **3**

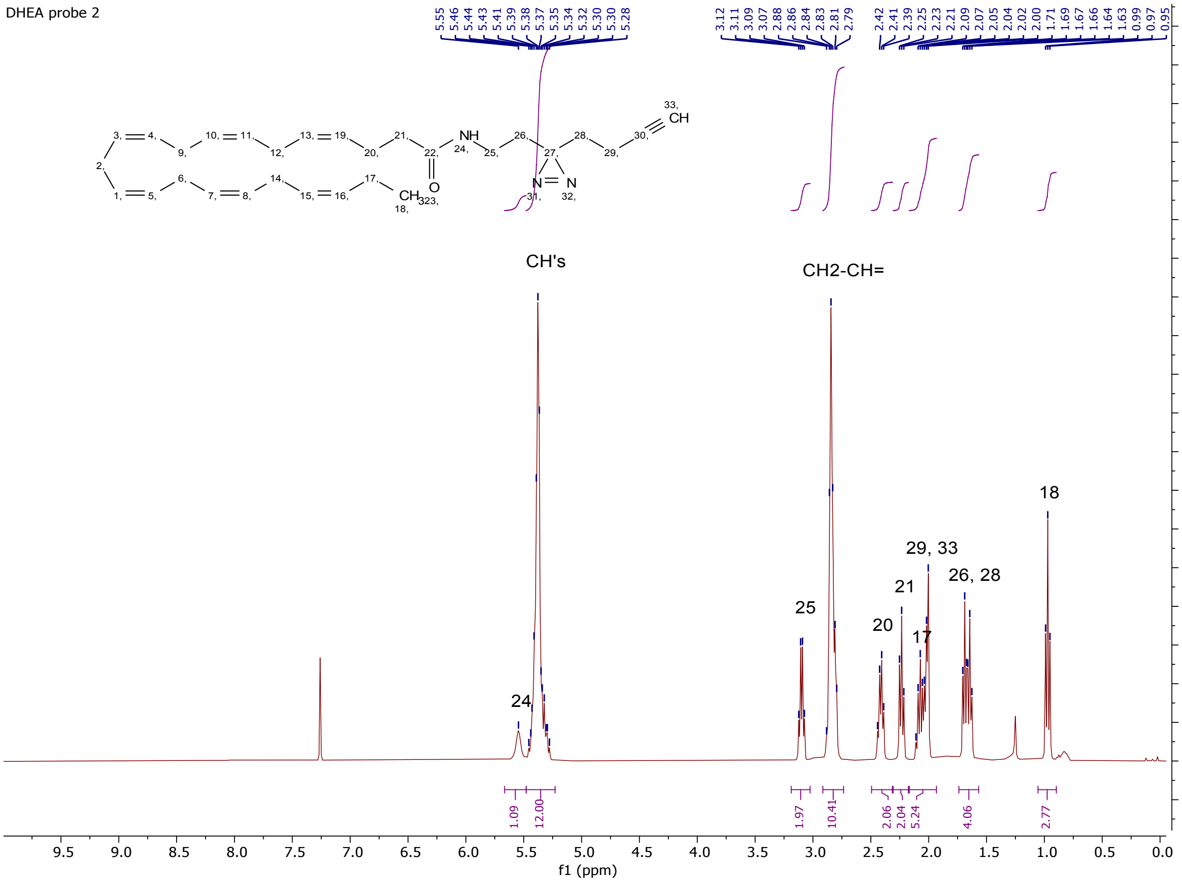

Supplementary Figure 12 ^{1}H-NMR spectrum of DHEA probe **2**

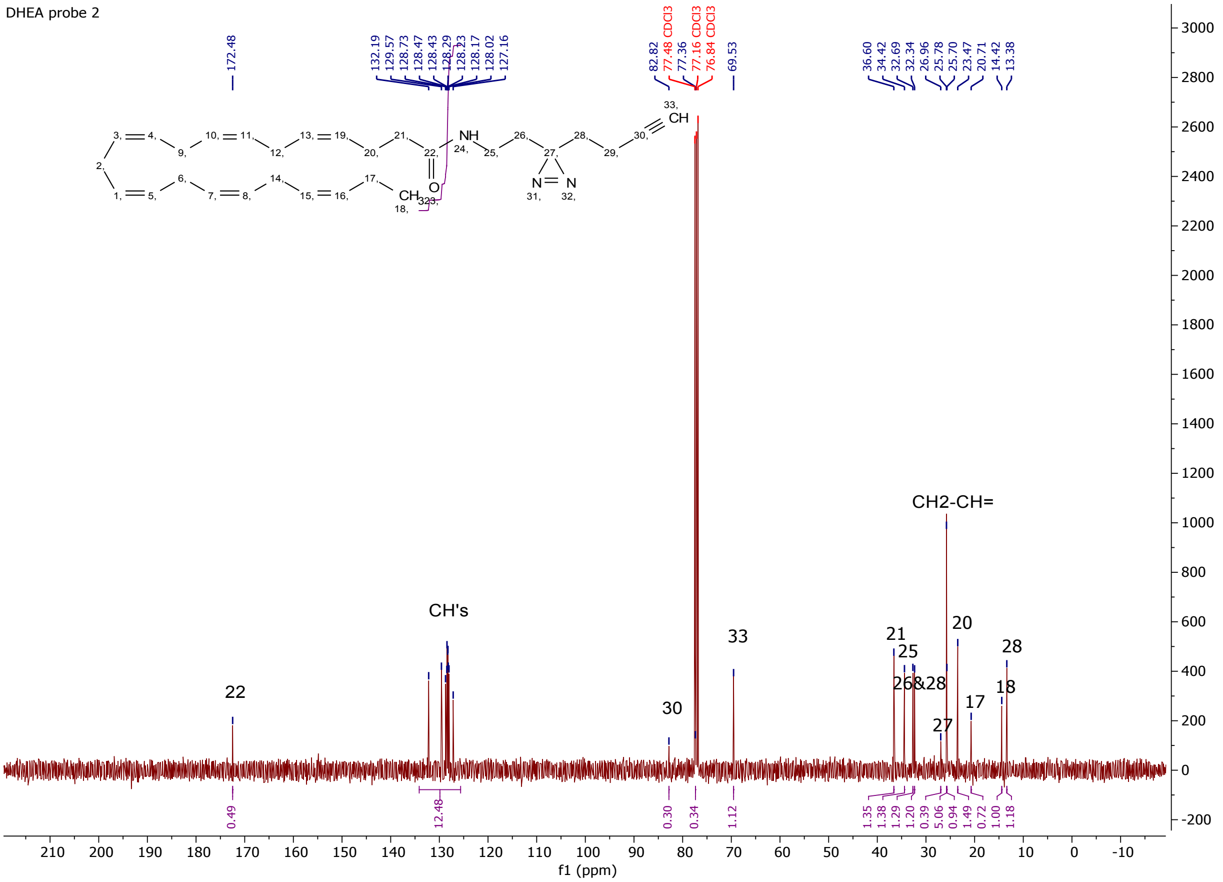

Supplementary Figure 13 ^{13}C NMR of DHEA probe **2**

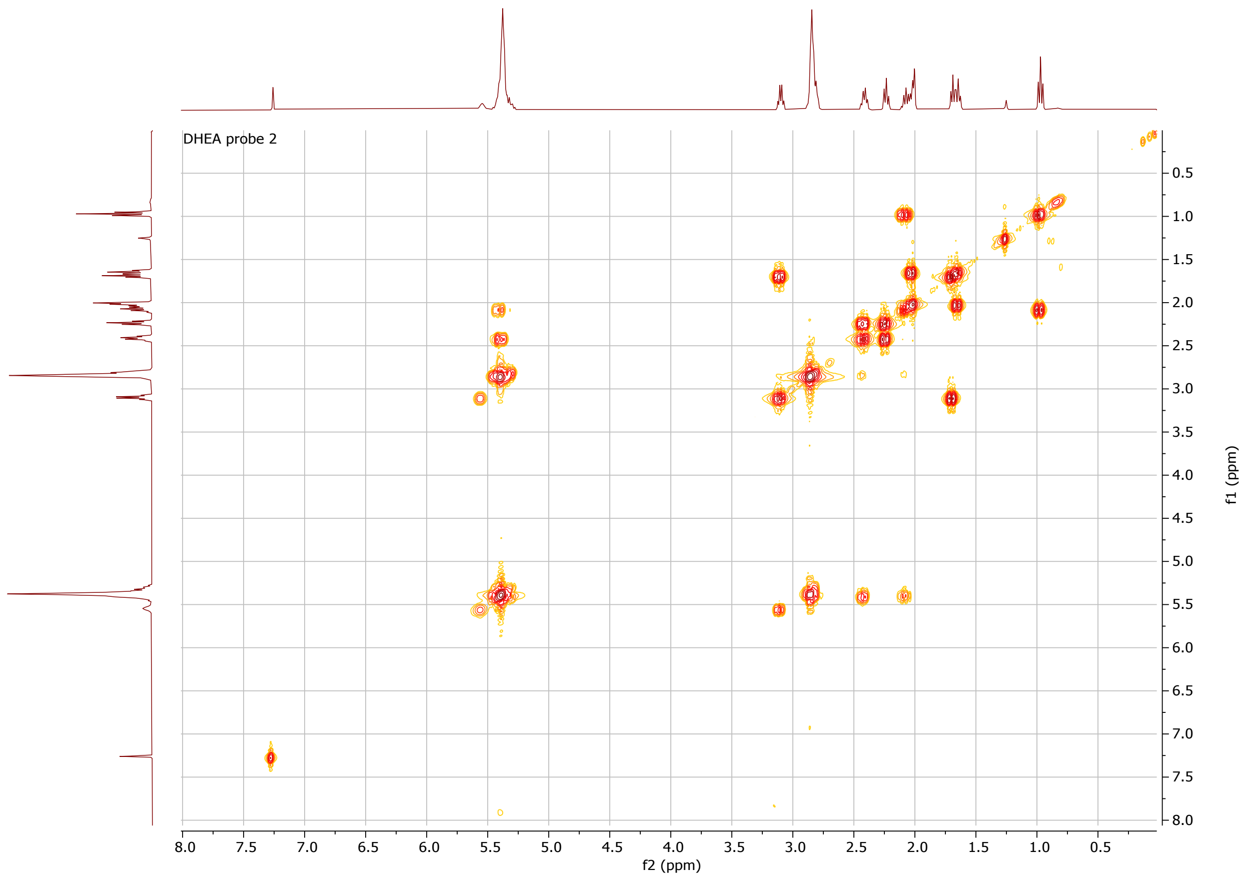

Supplementary Figure 14 COSY spectrum of DHEA probe **2**

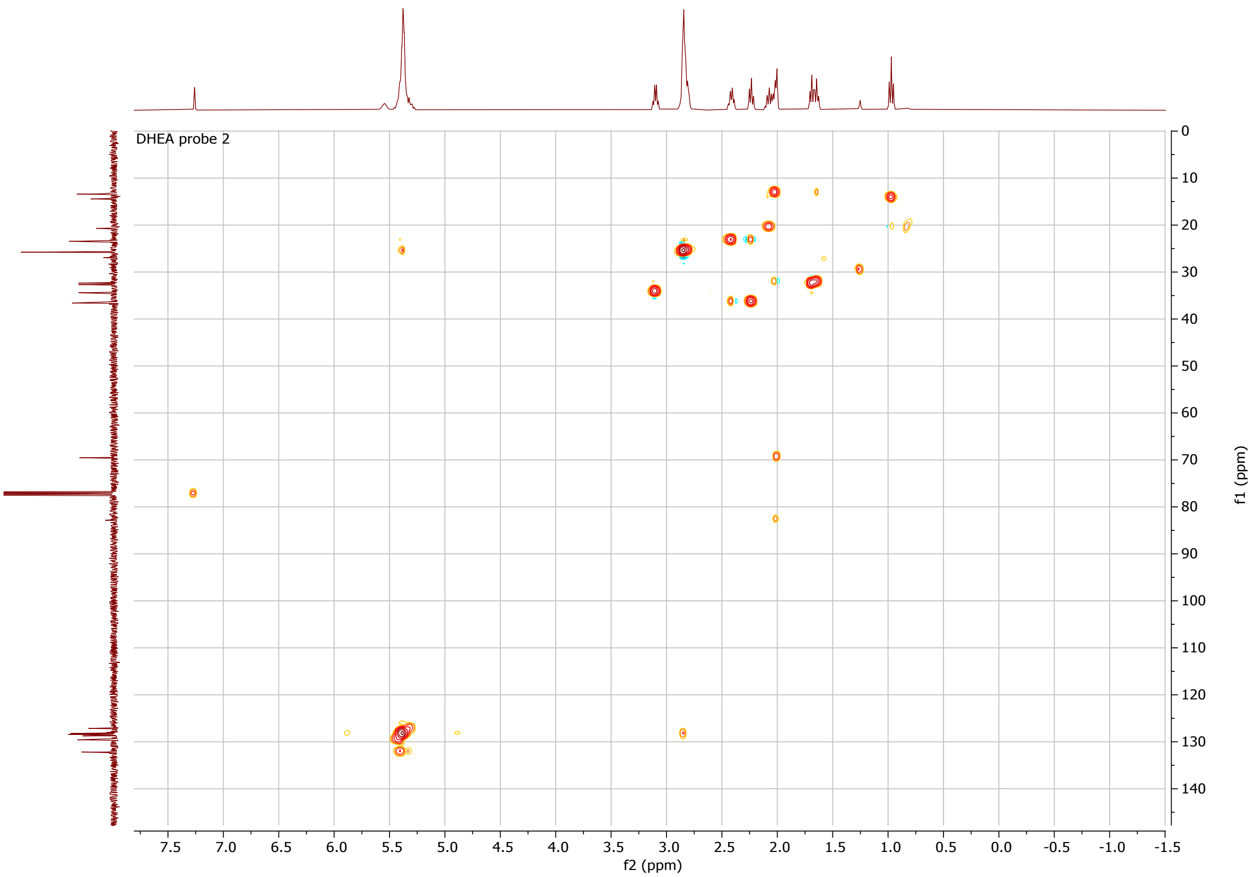

Supplementary Figure 15 HSQC spectrum of DHEA probe **2**

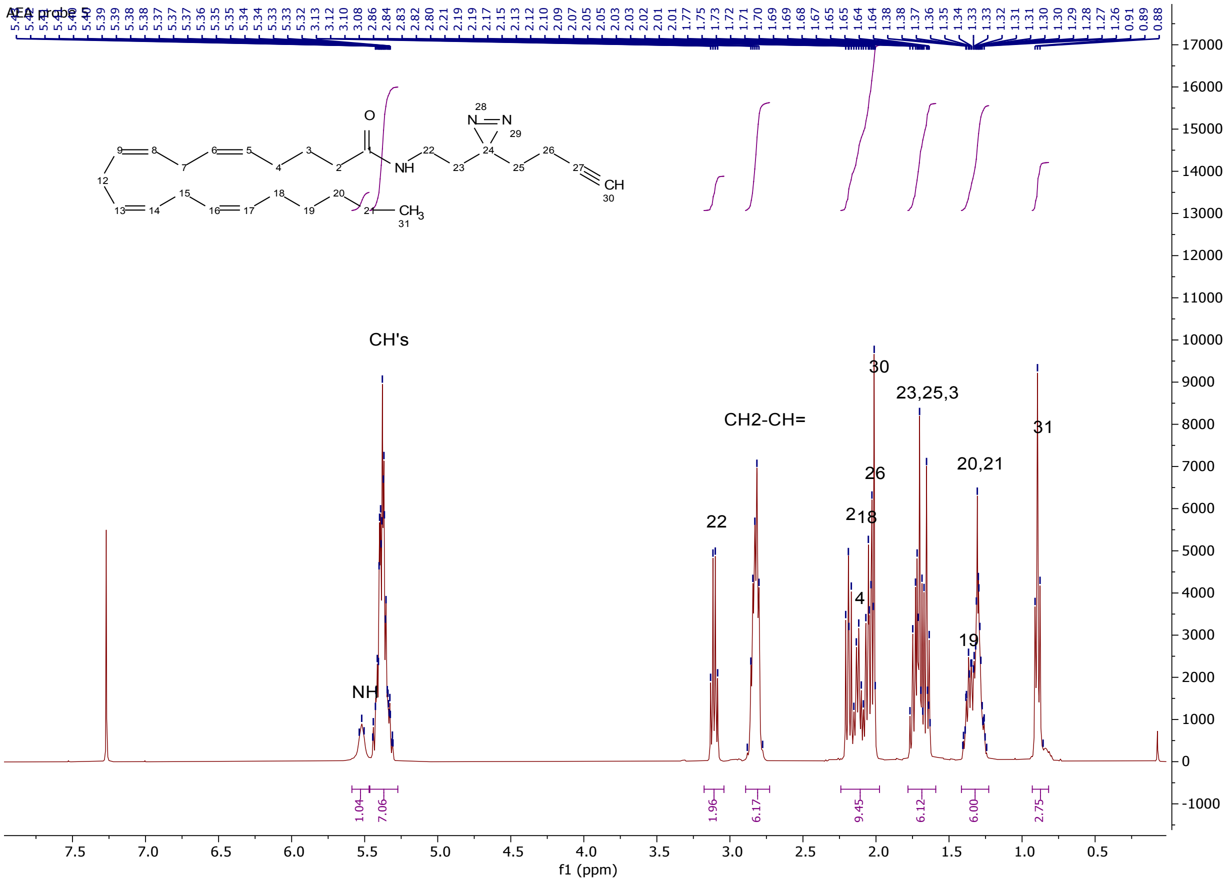

Supplementary Figure 16 ^{1}H-NMR spectrum of AEA probe **5**

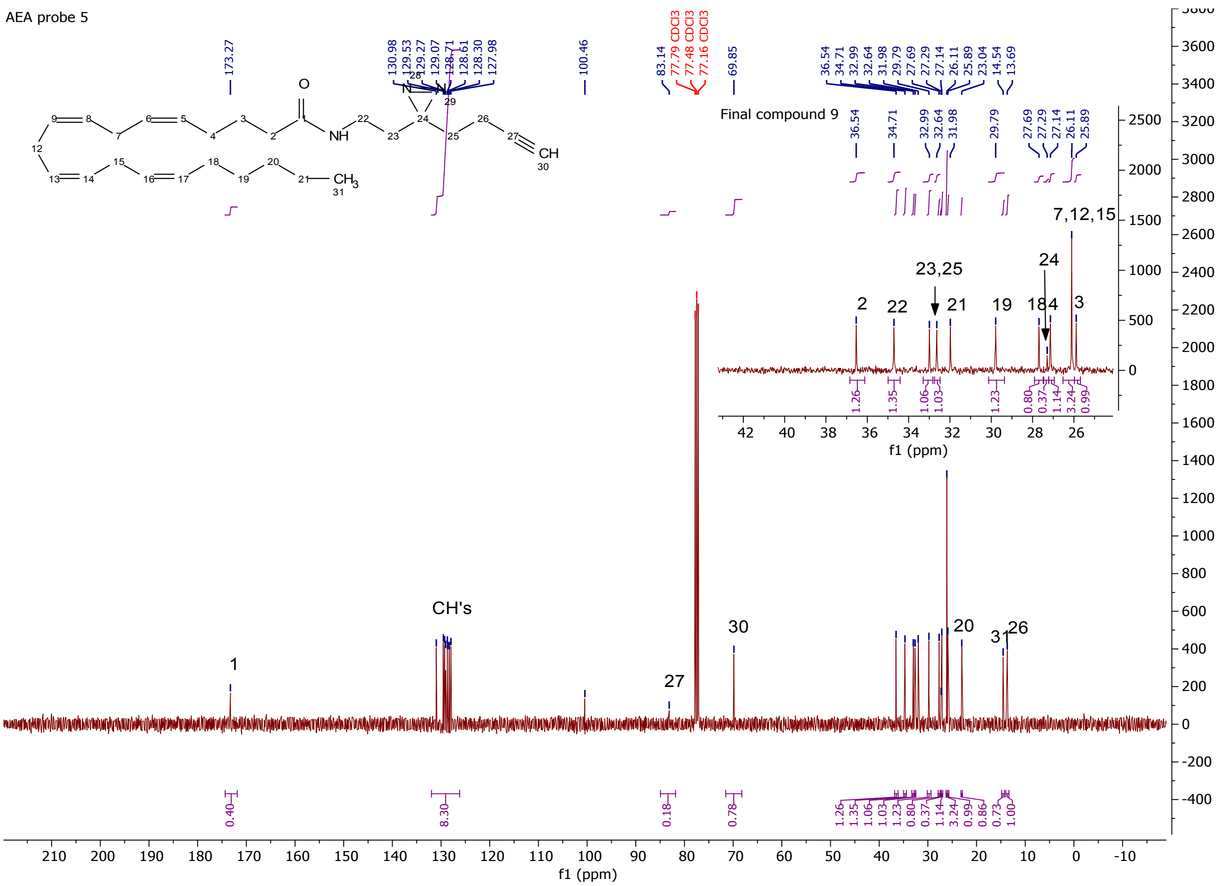

Supplementary Figure 17 ^{13}C NMR of AEA probe **5**

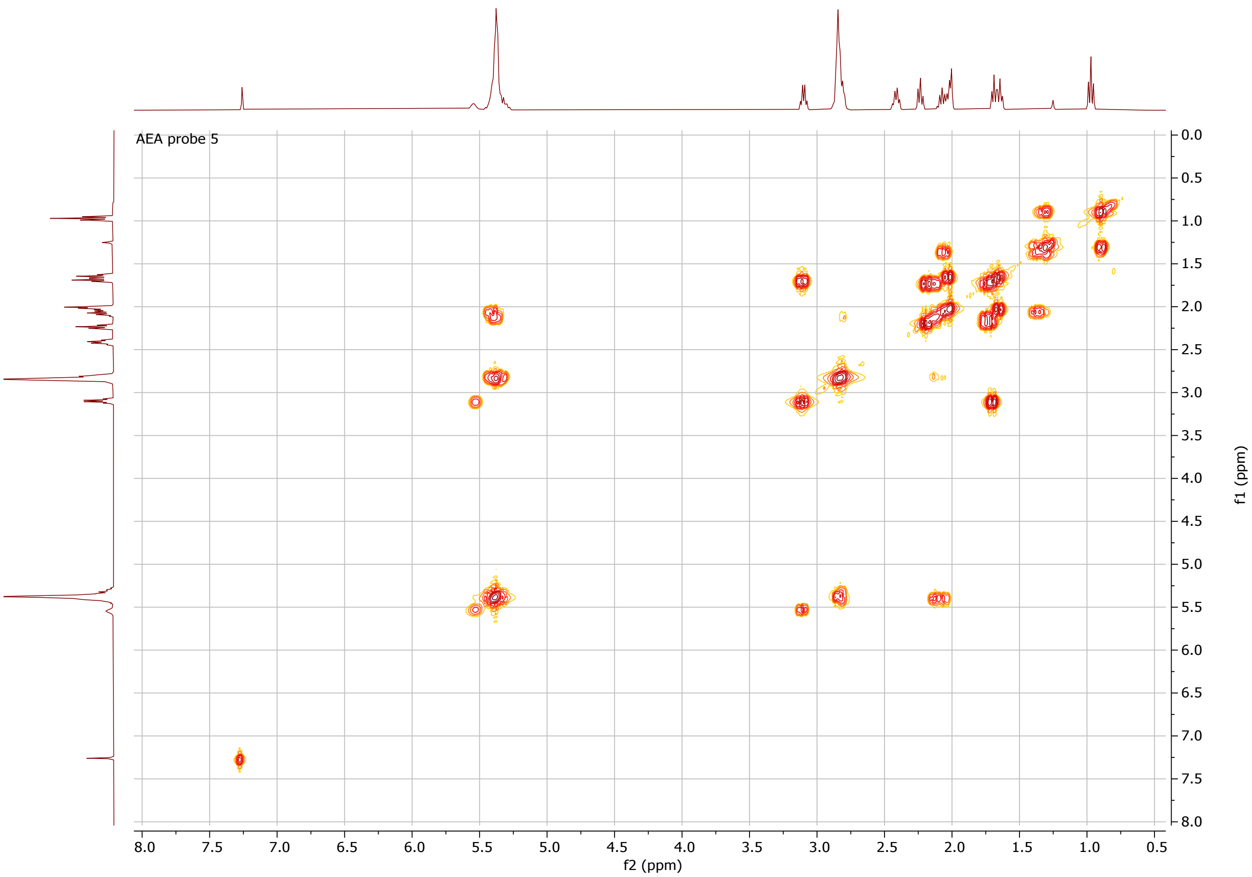

Supplementary Figure 18 COSY spectrum of AEA probe **5**

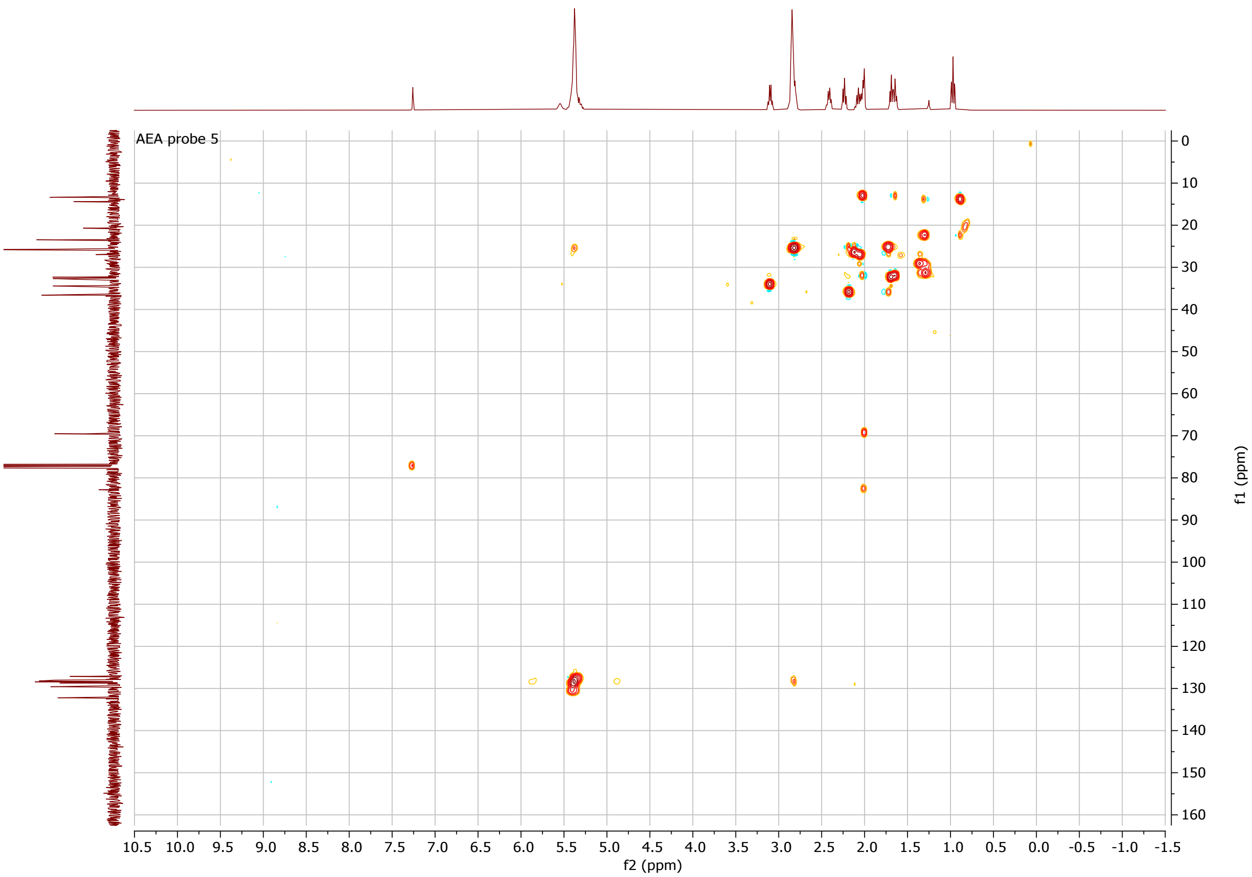

Supplementary Figure 19 HSQC spectrum of AEA probe **5**

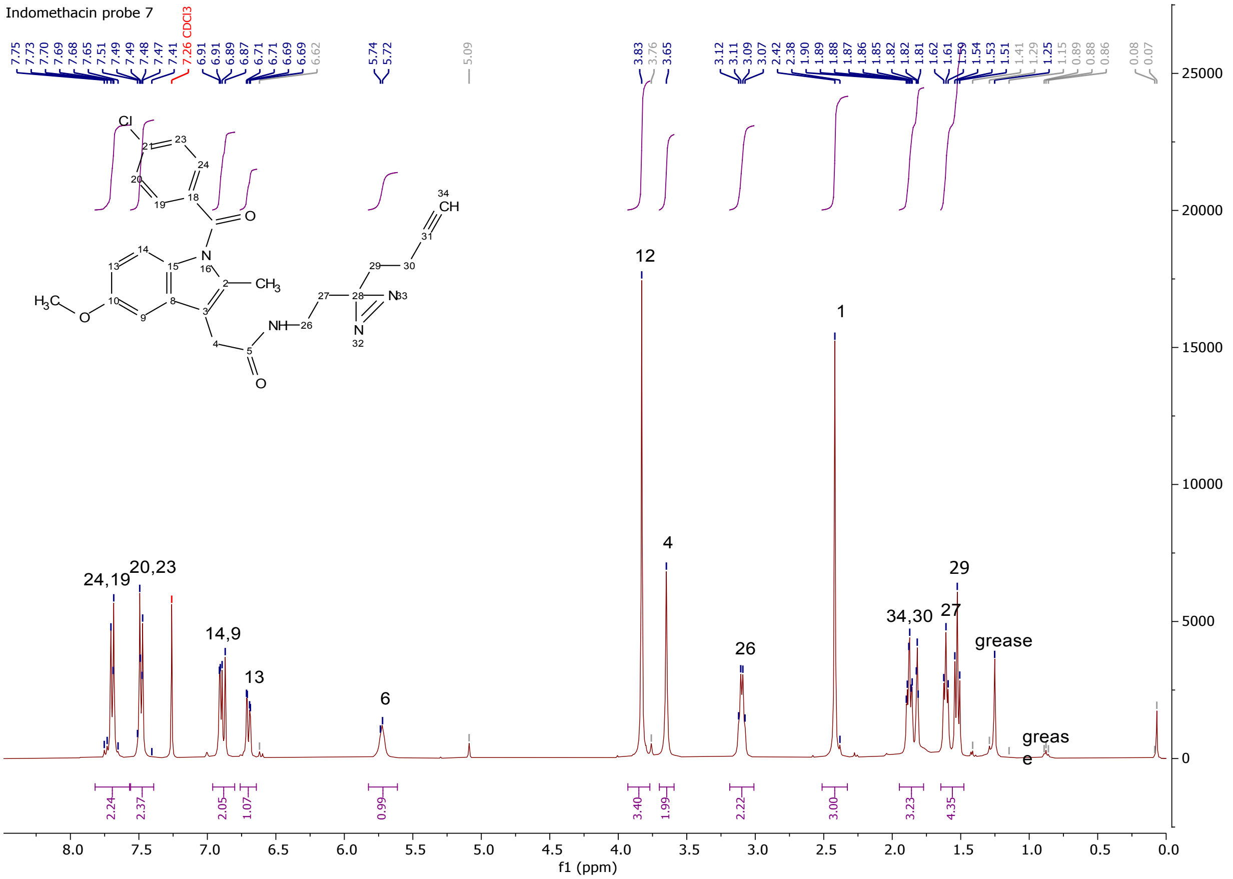

Supplementary Figure 20 ^{1}H-NMR spectrum of indomethacin probe **7**

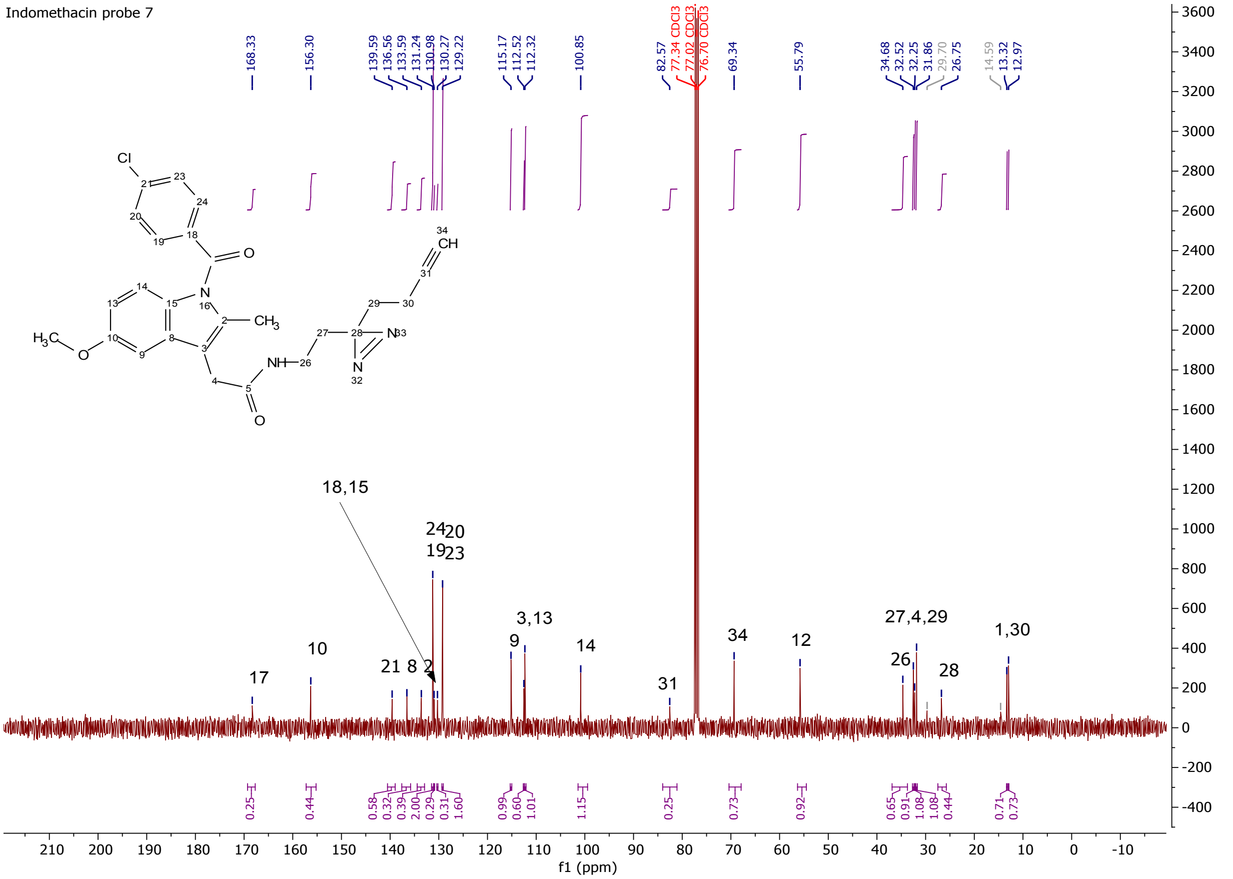

Supplementary Figure 21 ^{13}C-NMR spectrum of indomethacin probe **7**

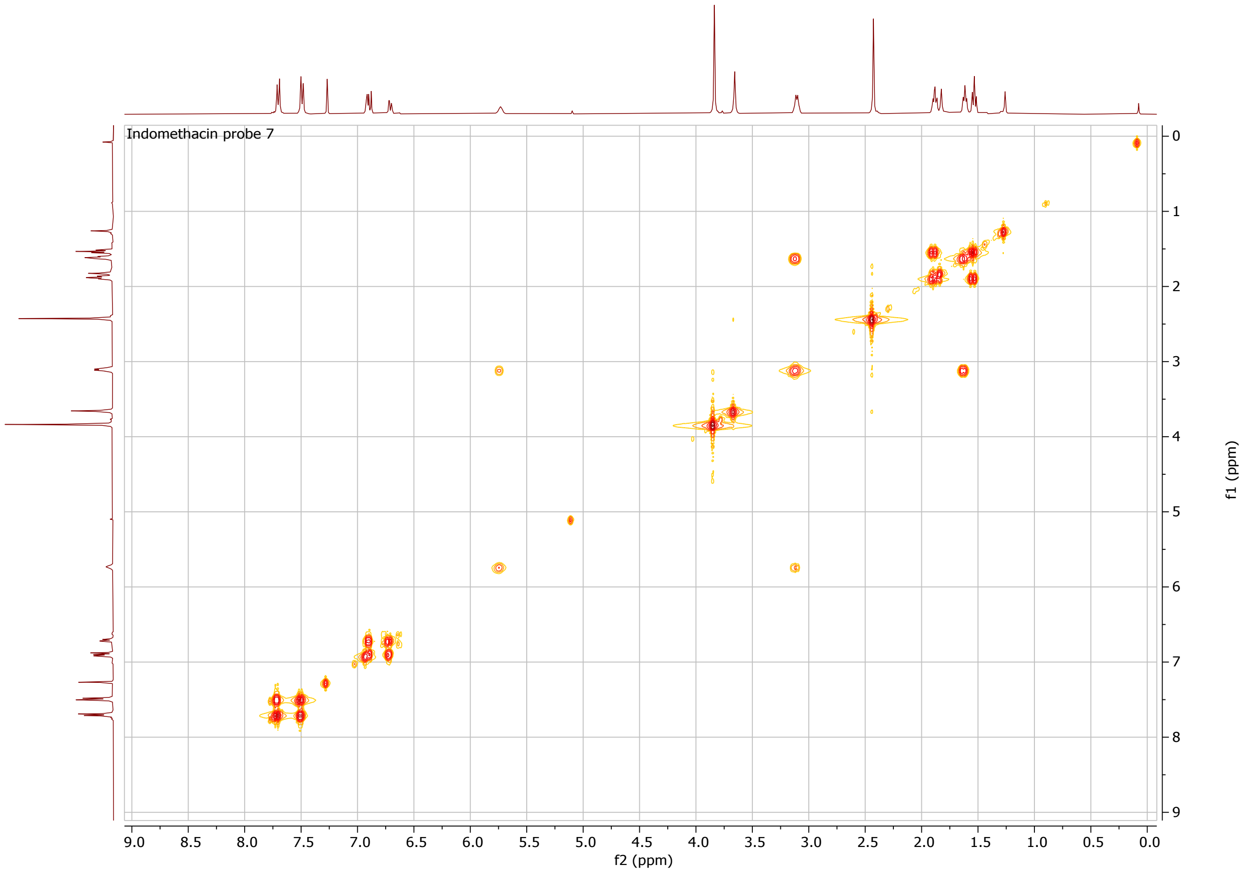

Supplementary Figure 22 COSY spectrum of indomethacin probe **7**

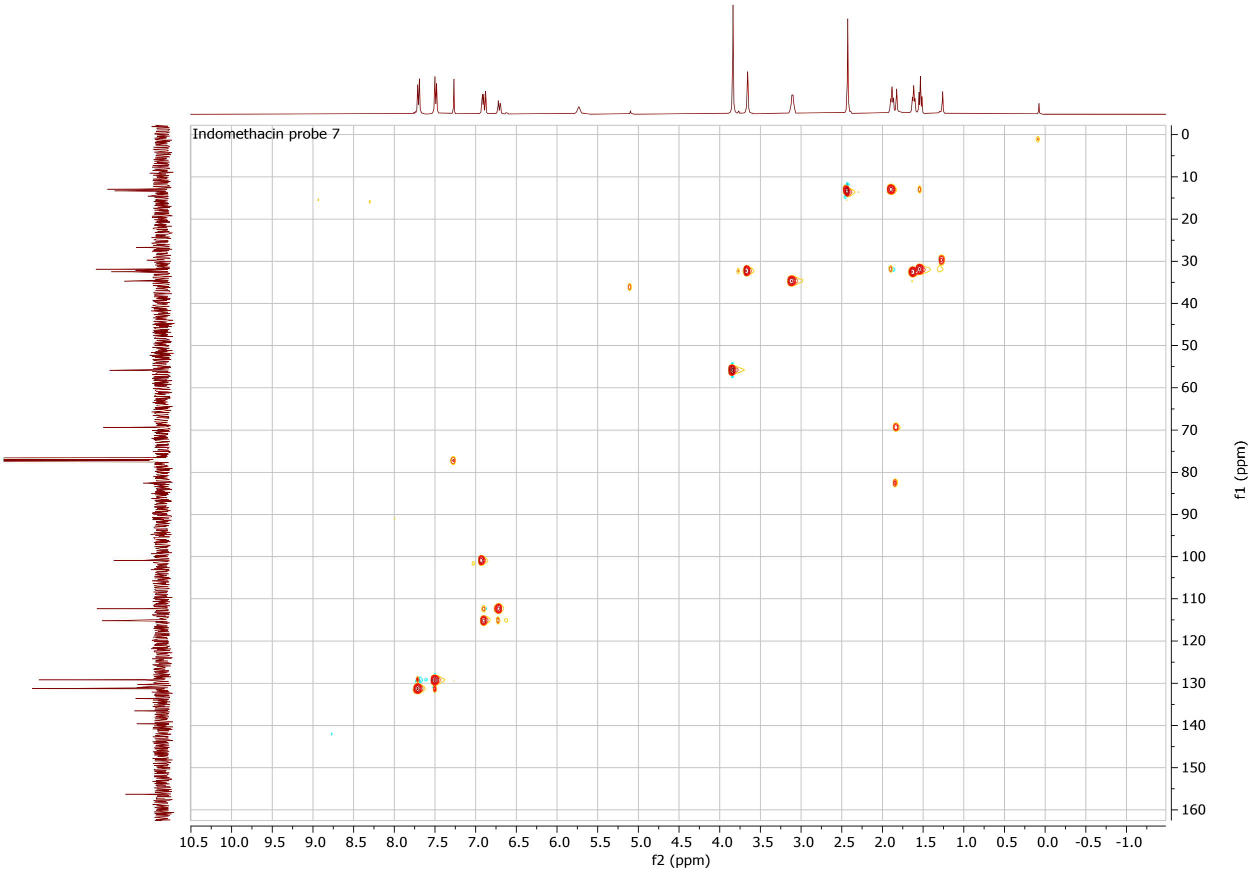

Supplementary Figure 23 HSQC spectrum of indomethacin probe **7**

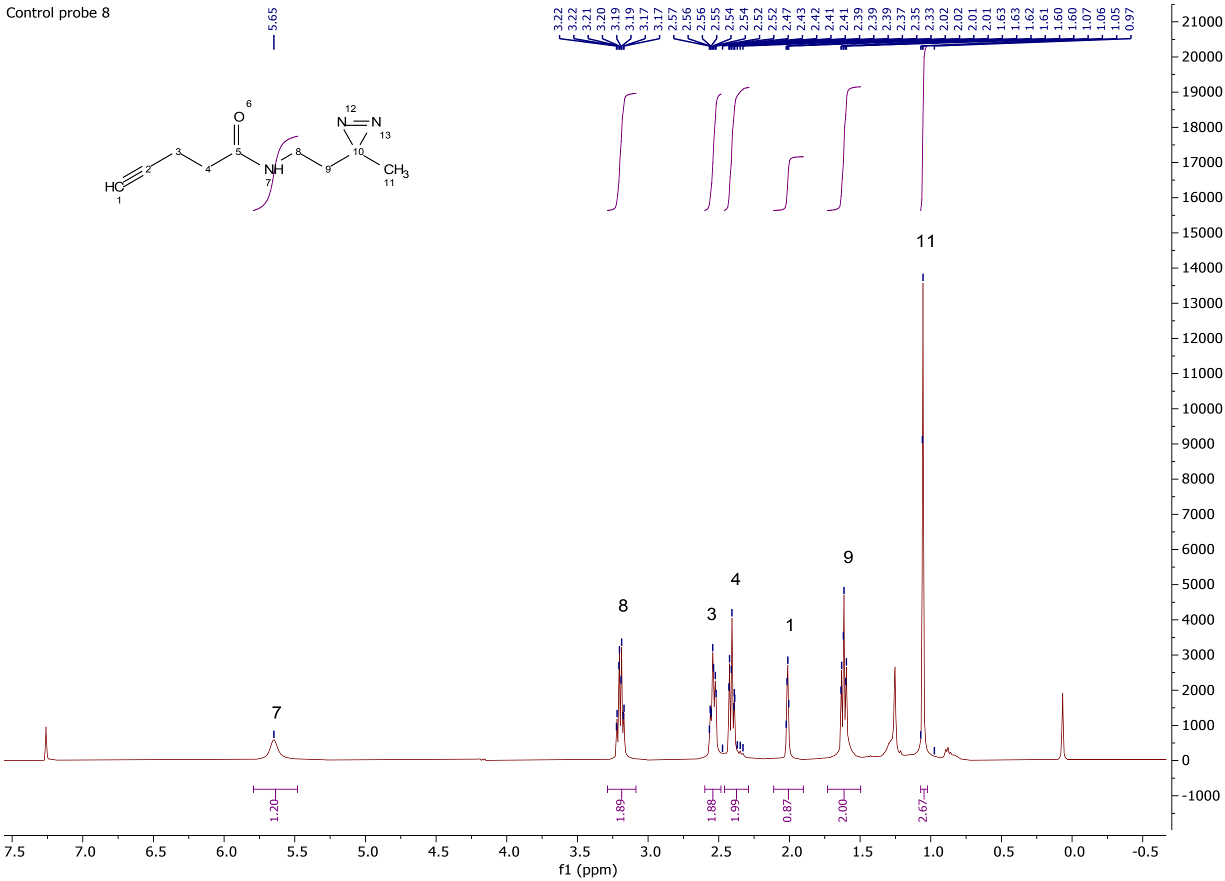

Supplementary Figure 24 ^{1}H-NMR spectrum of control probe **8**

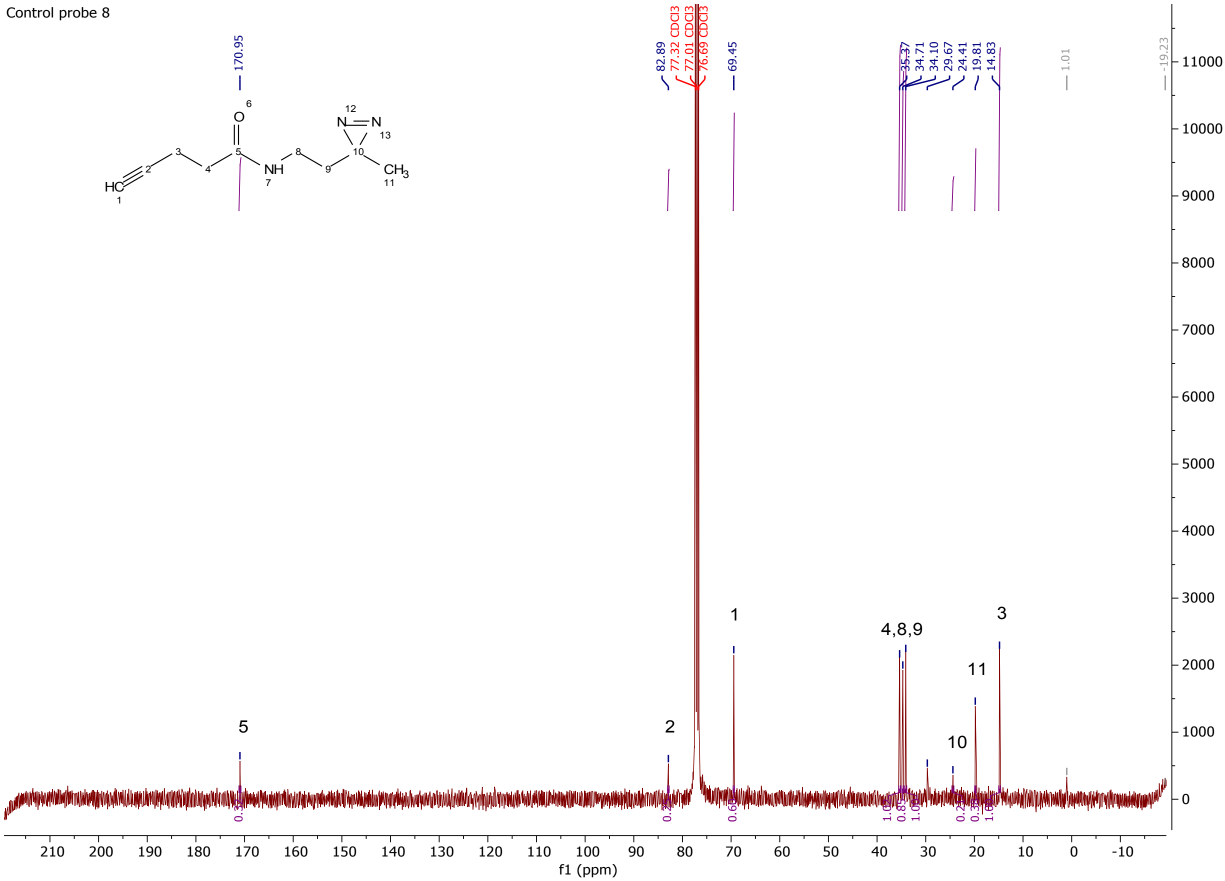

Supplementary Figure 25 ^{13}C spectrum of control probe **8**

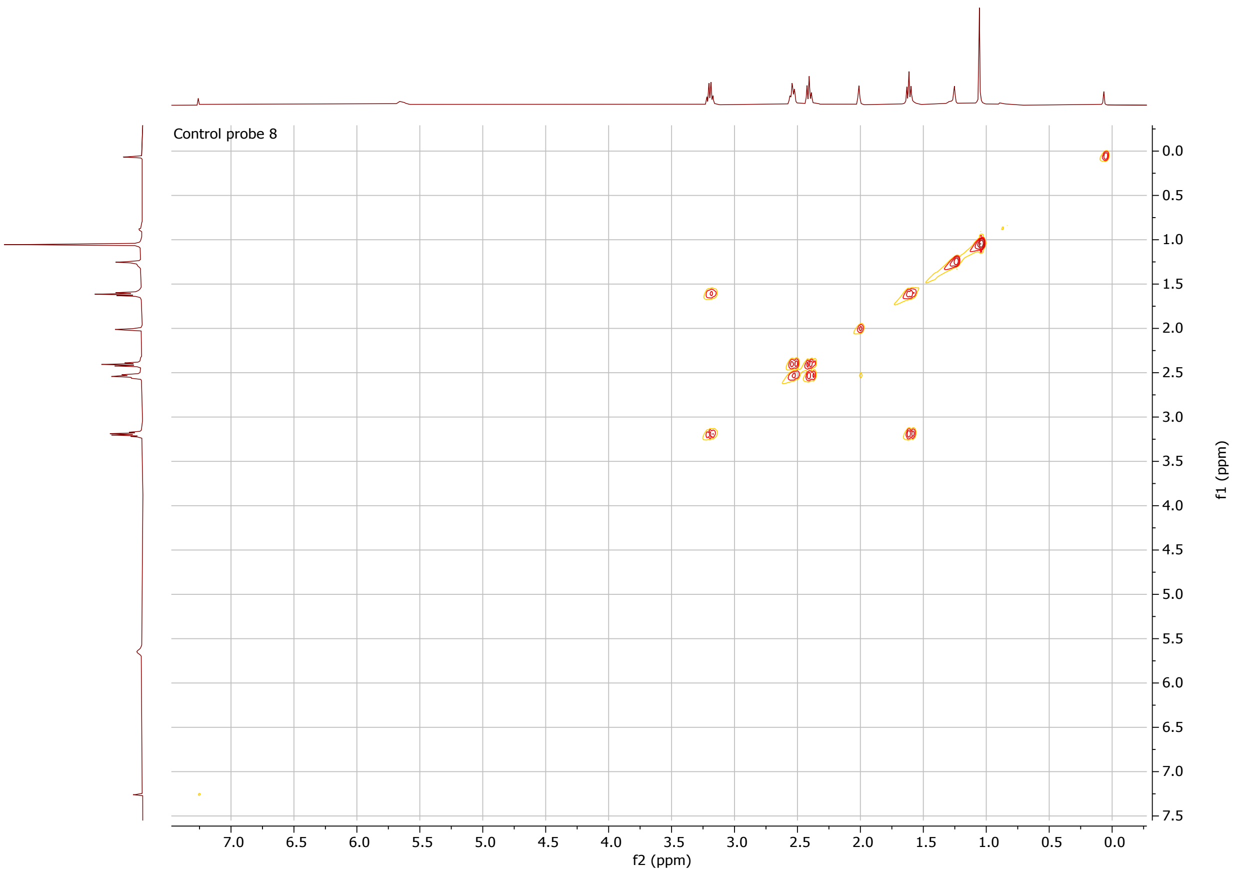

Supplementary Figure 26 COSY spectrum of control probe **8**

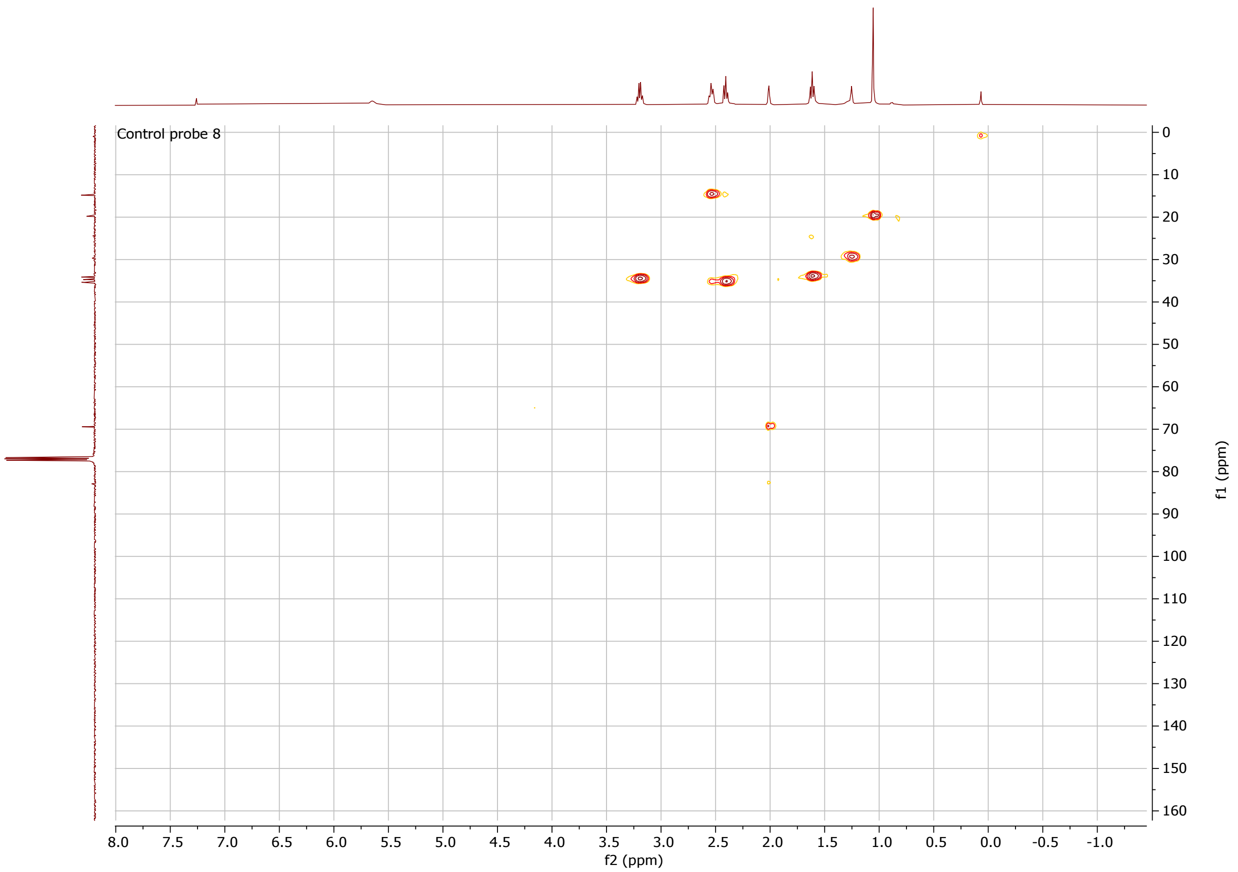

Supplementary Figure 27 HSQC spectrum of control probe **8**

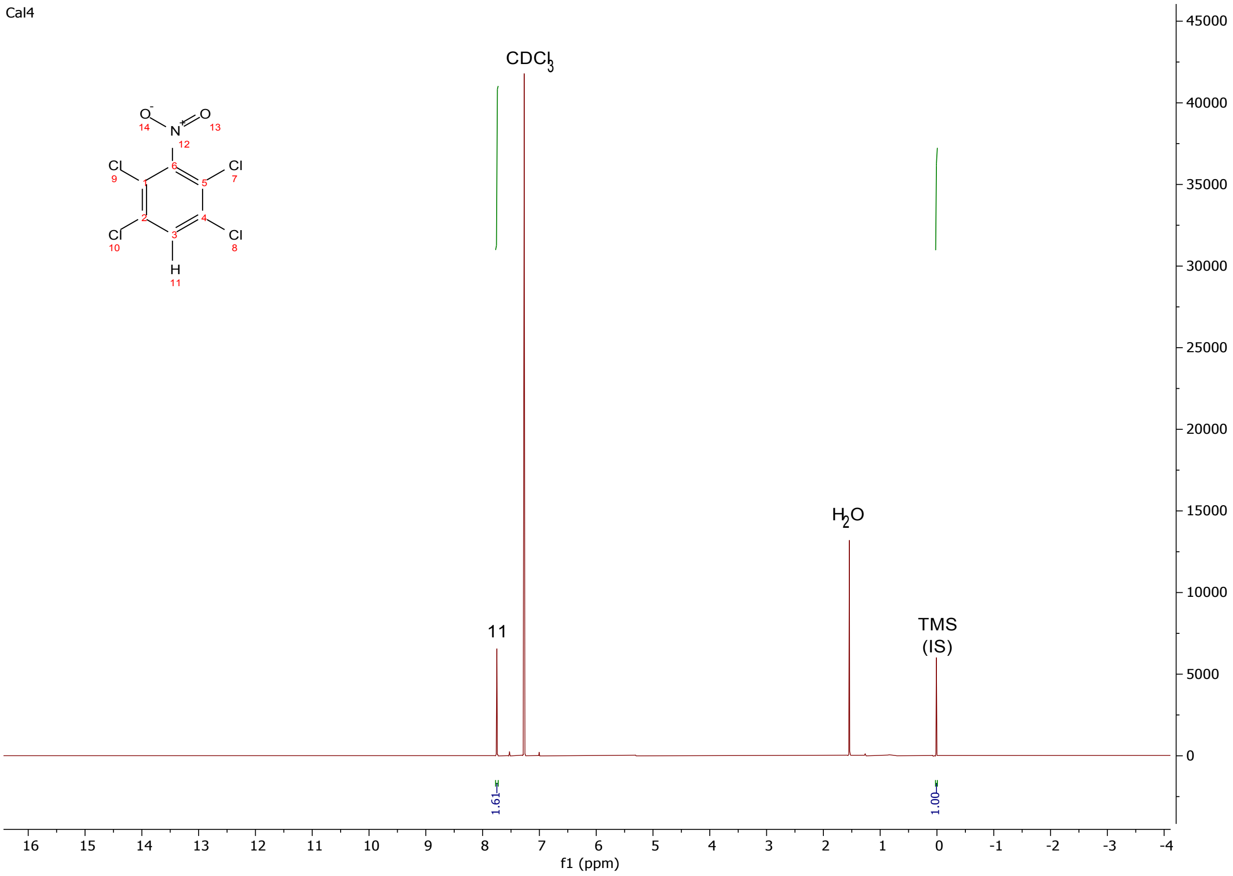

Supplementary Figure 28 ^{1}H NMR of 3.8 mM 1,2,4,5-Tetrachloro-3-nitrobenzene, used for the calibration in the quantification of DHEA probe **3**

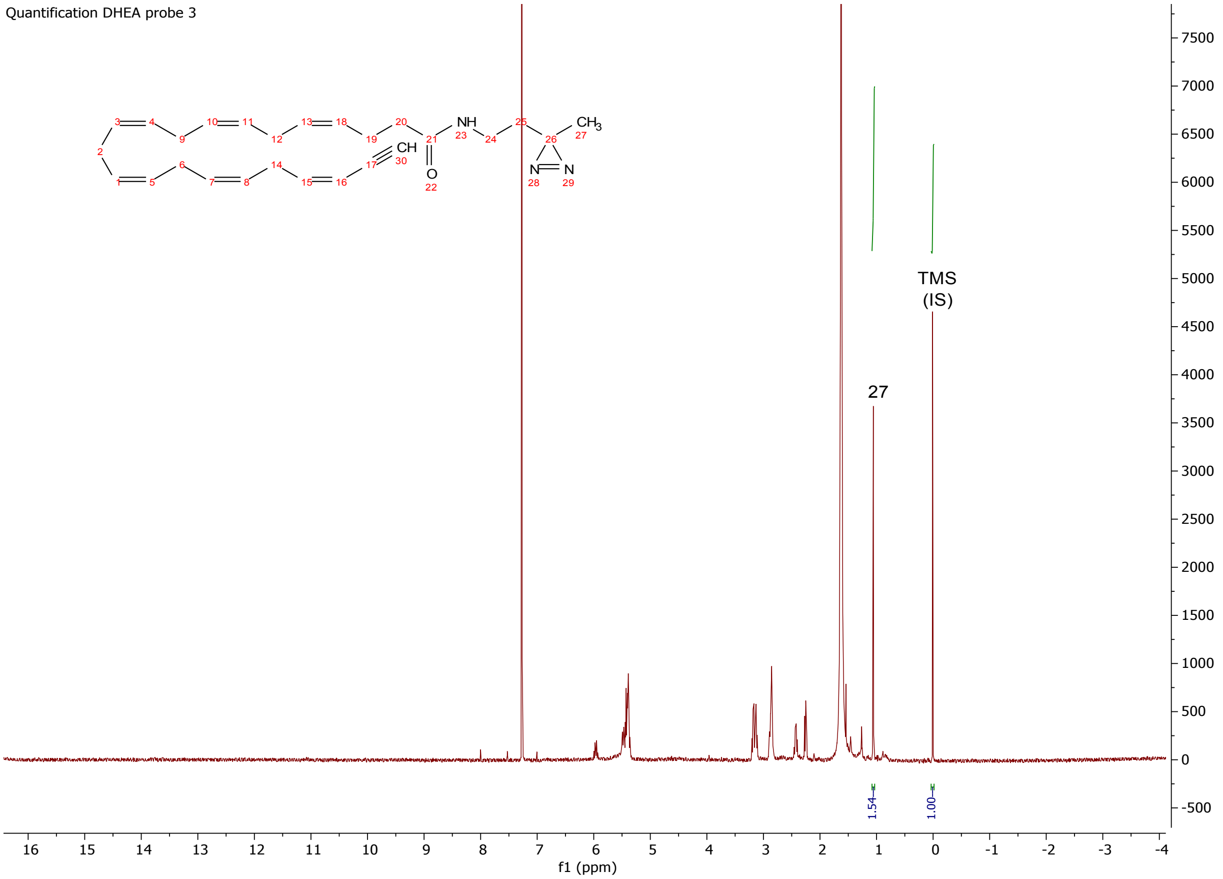

Supplementary Figure 29 ^{1}H NMR of the quantification run of DHEA probe **3**

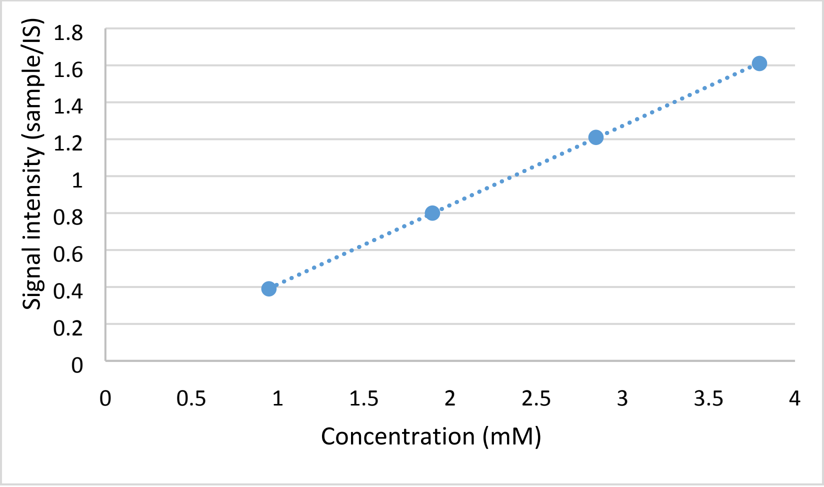

Supplementary Figure 30 Calibration curve of 1,2,4,5-Tetrachloro-3-nitrobenzene, used for the quantification of DHEA probe **3**

References

[1] Gaebler, A., Penno, A., Kuerschner, L., and Thiele, C. (2016) A highly sensitive protocol for microscopy of alkyne lipids and fluorescently tagged or immunostained proteins, *J. Lipid Res. 57*, 1934-1947.

[2] Schwanhäusser, B., Busse, D., Li, N., Dittmar, G., Schuchhardt, J., Wolf, J., Chen, W., and Selbach, M. (2011) Global quantification of mammalian gene expression control, *Nature 473*, 337-342.

[3] The UniProt, C. (2019) UniProt: a worldwide hub of protein knowledge, *Nucleic Acids Res. 47*, D506-D515.

[4] Thiele, C., Papan, C., Hoelper, D., Kusserow, K., Gaebler, A., Schoene, M., Piotrowitz, K., Lohmann, D., Spandl, J., Stevanovic, A., Shevchenko, A., and Kuerschner, L. (2012) Tracing Fatty Acid Metabolism by Click Chemistry, *ACS Chem. Biol. 7*, 2004-2011.
